# Recent emergence of Arctic atlantification dominated by climate warming

**DOI:** 10.1126/sciadv.adq5235

**Published:** 2024-11-27

**Authors:** Qiang Wang, Qi Shu, Fan Wang

**Affiliations:** ^1^ Alfred Wegener Institute, Helmholtz Centre for Polar and Marine Research (AWI), Bremerhaven, Germany.; ^2^ First Institute of Oceanography and Key Laboratory of Marine Science and Numerical Modeling, Ministry of Natural Resources, Qingdao, China.; ^3^ Shandong Key Laboratory of Marine Science and Numerical Modeling, Qingdao, China.; ^4^ Key Laboratory of Ocean Observation and Forecasting and Key Laboratory of Ocean Circulation and Waves, Institute of Oceanology, Chinese Academy of Sciences, Qingdao, China.

## Abstract

The Arctic Ocean’s Eurasian Basin underwent notable atlantification during the 2010s, characterized by warming of the Atlantic Water layer and increased upper ocean salinity. Despite profound implications for the Arctic climate system and marine ecosystems, the primary drivers of this process remain debated. One hypothesis suggested that alternating phases of the atmospheric Arctic Dipole may have mitigated recent atlantification. Here, we use high-resolution model simulations to disentangle the main contributors to atlantification in the Arctic basin. We show that the decline in Arctic sea ice was the dominant driver, while wind variability associated with the Arctic Dipole played a minor role, contributing slightly rather than mitigating the process. The positive phase of the Arctic Oscillation also made a relatively small contribution. Although recent changes in atmospheric circulation over the Greenland Sea tended to reduce warm water inflow through the Fram Strait, this cooling effect on the Arctic Atlantic Water layer was outweighed by the warming induced by sea ice decline.

## Teaser

Arctic atlantification emerged with contributions from natural variability, but climate change was the primary driver.

## INTRODUCTION

The Arctic region is undergoing rapid changes, with near-surface air temperature rising three to four times faster than the global average (*1*, *2*). Concurrently, there has been a notable reduction in both the extent and thickness of Arctic sea ice (*3*, *4*). Moreover, the Arctic Ocean is experiencing remarkable changes. Over the past two decades, there has been a substantial accumulation of liquid fresh water in the Beaufort Gyre region (*5*–*8*). During the 2010s, the Arctic Eurasian Basin witnessed a warming trend in the Atlantic Water layer, along with increased salinity and weakened stratification in the overlying halocline—phenomena collectively known as Arctic atlantification (*9*).

The Arctic Ocean plays an important role in the hydrological cycle of the Northern Hemisphere, with its storage and release of fresh water potentially influencing the formation of dense waters in the subpolar North Atlantic, thus affecting large-scale ocean circulation and climate (*10*–*12*). The warming of the Arctic Ocean and the weakening in its halocline stratification may contribute to the basal melting of sea ice, creating a feedback loop that accelerates Arctic sea ice decline (*13*, *14*). The Arctic Ocean also harbors a marine ecosystem adapted to its unique climate conditions, making it particularly vulnerable to ongoing changes (*15*, *16*). Therefore, gaining a comprehensive understanding of the primary drivers behind the observed changes in the Arctic Ocean is of critical importance.

The Arctic Atlantic Water layer is mainly supplied by the Atlantic Water inflow through the Fram Strait (fig. S1), whose volume transport and temperature can be increased by strengthened cyclonic gyre circulation in the Greenland Sea (*17*, *18*). The strength of this gyre circulation is influenced by changes in local winds and salinity changes associated with the export of sea ice fresh water through the Fram Strait, thereby affecting the Atlantic Water inflow (*19*, *20*). In addition, strengthening in the cyclonic ocean circulation in the Eurasian Basin can also enhance Fram Strait inflow (*18*, *21*). A recent study proposed that the second leading mode of the Arctic atmospheric circulation, known as the Arctic Dipole (*22*), played a crucial role in modulating the Arctic atlantification through changing the Atlantic Water inflow (*23*). According to this study, natural climate variability, specifically linked to the decadal alternation in the Arctic Dipole, may have weakened the northward warm water inflow through the Fram Strait and transferred liquid fresh water from the Eurasian Basin to the Amerasian Basin during the 2010s. Atlantification has also been observed in the Barents Sea (*15*, *24*), attributed to both warming of the Atlantic Water inflow through the Barents Sea Opening and reduced ocean surface heat loss in the southwestern Barents Sea (*25*–*27*).

The explanations for the freshwater accumulation and state transition of the Beaufort Gyre during the 2010s vary widely. These range from the phase change of the Arctic Oscillation, the first leading mode of the Arctic atmospheric circulation (*28*), to the impacts of the Arctic Dipole and sea ice decline (*23*, *29*, *30*). The sources of Beaufort Gyre fresh water include Pacific Water from the Bering Strait, river runoff, sea ice meltwater, and net precipitation, all of which are currently increasing in a warming climate (*7*, *8*, *21*, *31*), potentially contributing to Arctic freshwater accumulation.

Despite the importance of the atlantification in the Arctic deep basin and the concurrent changes in freshwater spatial distribution, there is still no consensus on their main drivers. Here, we use dedicated numerical simulations to unravel the primary factor responsible for the Arctic atlantification in the Eurasian Basin in the 2010s. We find that its occurrence was predominantly driven by sea ice decline. Wind forcing associated with recent positive phases of the Arctic Dipole and Arctic Oscillation also contributed to the atlantification but with a relatively small role.

## RESULTS

### Sea ice decline and changes in atmospheric circulation

As our study investigates how changes in Arctic sea ice and winds contributed to the emergence of atlantification, we begin by briefly reviewing these changes. Since the beginning of satellite observations, the Arctic has experienced a marked decline in both sea ice extent and volume (fig. S2). Over the satellite era, summer sea ice extent has decreased by approximately half, and by the 2010s, mean sea ice thickness at the end of the melt season had dropped by more than 60% compared to six decades ago (*3*, *4*). Moreover, during the first two decades of the 21st century, the coverage of multiyear sea ice in the Arctic has declined by over 50% (*3*).

**Fig. 1.**
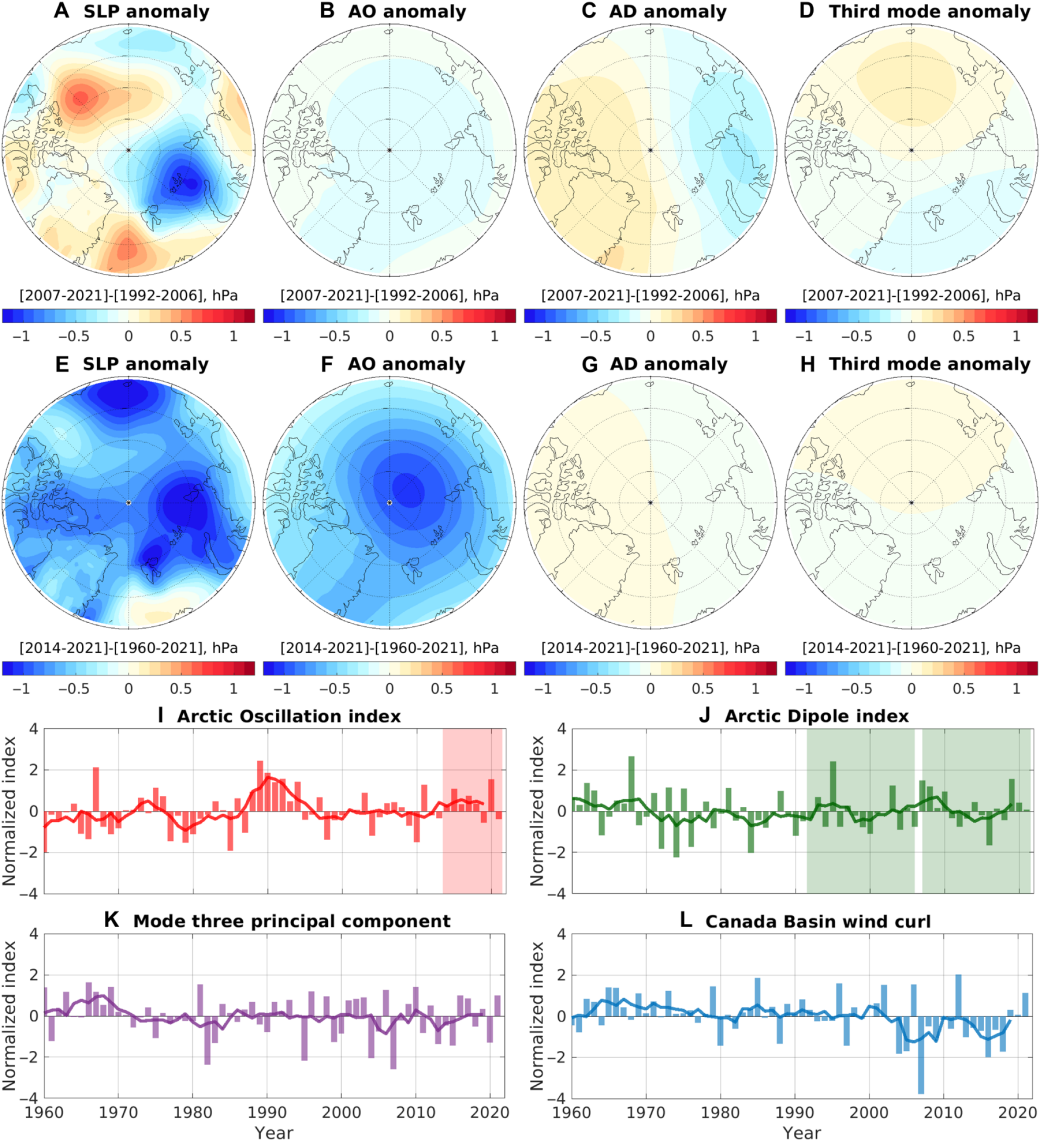
Recent changes in atmospheric circulation. (**A**) SLP anomaly between the period of 2007 to 2021 and the period of 1992 to 2006. (**B** to **D**) Contribution of the (B) Arctic Oscillation, (C) Arctic Dipole, and (D) the third mode to the SLP anomaly between these two periods. (**E**) SLP anomaly between the period of 2014 to 2021 and the long-term (1960 to 2021) mean. (**F** to **H**) Contribution of the (F) Arctic Oscillation, (G) Arctic Dipole, and (H) the third mode to the SLP anomaly between these two periods. (**I**) Arctic Oscillation index. (**J**) Arctic Dipole index. (**K**) Index of the third mode of the Arctic atmospheric circulation. (**L**) Wind curl over the Canada Basin. Bars denote normalized annual mean values, and solid lines denote their 5-year running means.

The sea level pressure (SLP) difference between the periods of 2007 to 2021 and 1992 to 2006 shows a tripole pattern (Fig. 1A). This pattern features a negative SLP (indicating cyclonic atmospheric circulation) anomaly in the Eurasian Arctic, along with positive SLP (indicating anticyclonic atmospheric circulation) anomalies over the Canada Basin and Greenland Sea. The positive SLP anomaly over the Canada Basin is consistent with the predominantly negative wind curl anomalies in the region during the past two decades (Fig. 1L).

While a recent study (*23*) attributed the SLP anomaly between these two periods to decadal variability associated with the Arctic Dipole, it is noteworthy that their spatial patterns exhibit distinct characteristics (compare Fig. 1A and fig. S3B). Specifically, differences are observed in the locations of active centers and the presence or absence of an anticyclonic circulation anomaly over the Greenland Sea. To further investigate, we reconstructed the time series of SLP associated with the first three leading modes of the Arctic atmospheric circulation by multiplying each mode (fig. S3, A to C) with its corresponding principal component (Fig. 1, I to K). Subsequently, we calculated the SLP anomaly between the periods of 2007 to 2021 and 1992 to 2006 associated with each mode. Our results indicate that the SLP anomaly between these two periods cannot be explained by the decadal variability associated with any of these modes, including the Arctic Dipole (compare Fig. 1A and Fig. 1, B to D).

The difference in the Arctic Dipole index between the two periods exhibits considerable seasonal variation (fig. S4A). For instance, the index for the season from April to July is noticeably higher in the latter period (fig. S5A), while the difference for the annual mean index between the two periods is much smaller (fig. S5B). For understanding wind-driven ocean changes, it is necessary to consider the SLP anomaly with all seasons taken into account, as depicted in Fig. 1 (A to D).

The Arctic Oscillation entered a positive phase beginning in 2014 (Fig. 1I), with main contributions from winter (fig. S4B). The overall negative SLP anomaly in the period of 2014 to 2021 relative to the long-term mean (Fig. 1E) is consistent with the positive phase of the Arctic Oscillation (Fig. 1F). Moreover, the tripole pattern illustrated in Fig. 1A is also evident in the SLP anomaly for the period of 2014 to 2021 (Fig. 1E). In the following, we will use numerical simulations to unravel impacts of sea ice decline and different wind forcings corresponding to the SLP anomalies described above.

### Freshwater changes in the Arctic Ocean

To assess the distinct impacts of different factors on the Arctic Ocean, including the Arctic Dipole anomaly (Fig. 1C), the Arctic Oscillation anomaly (Fig. 1F), the overall SLP anomaly (Fig. 1A), and the decline in Arctic sea ice (fig. S2), we conducted four sensitivity simulations. Each simulation focused on isolating the effect of one specific factor by eliminating it from the model setup. Specifically, we performed three wind-perturbation experiments wherein the wind corresponding to the three SLP anomalies was subtracted from the wind forcing. In addition, we conducted an experiment wherein the Arctic surface thermal forcing was replaced with its climatology, effectively removing the declining trend of Arctic sea ice (refer to Materials and Methods). By comparing these sensitivity simulations with the control simulation—a historical simulation that represents past changes in ocean and sea ice—we can quantify the individual influence of each factor.

**Fig. 2.**
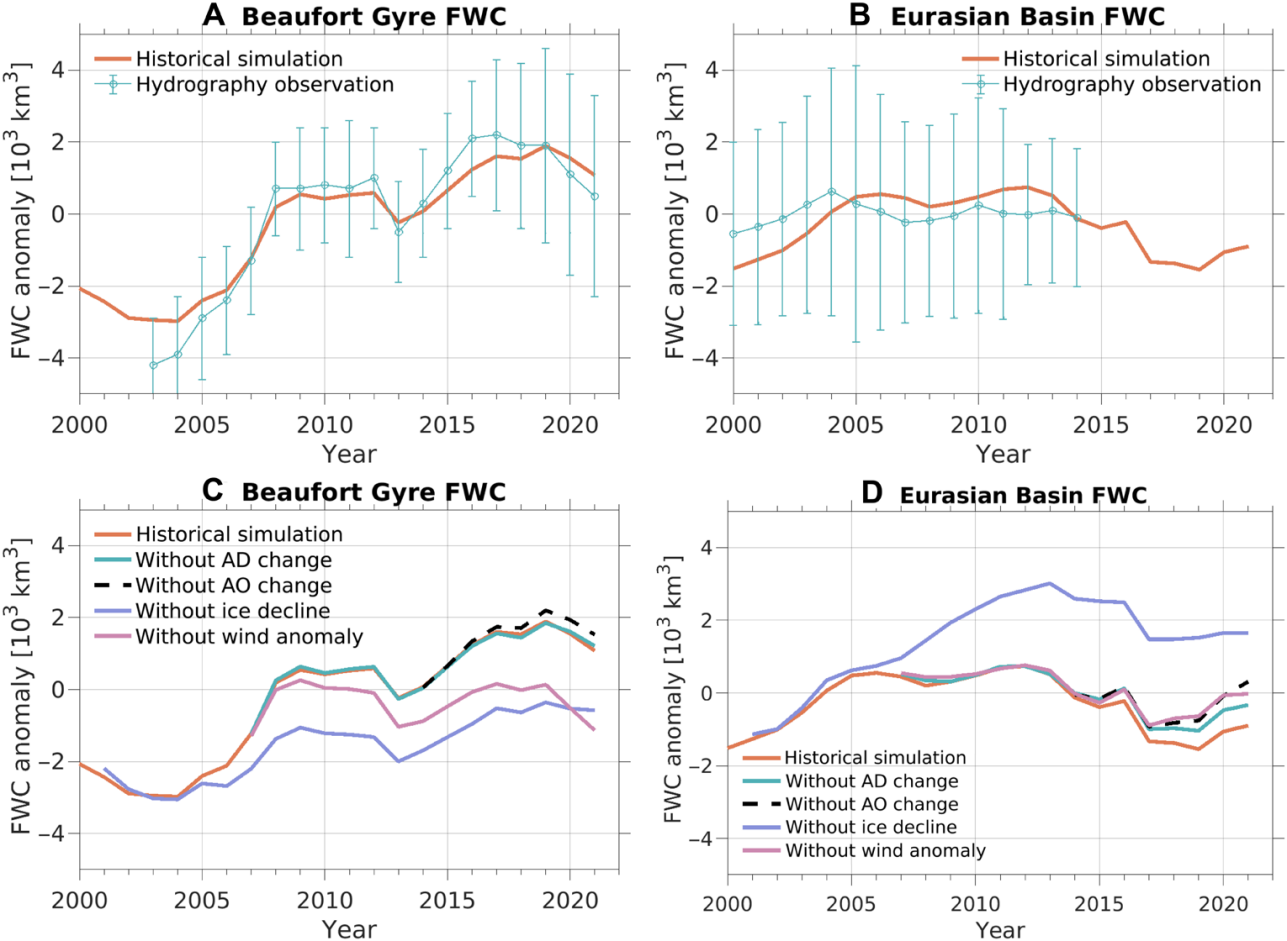
Liquid FWC and impacts of winds and sea ice decline. (**A** and **B**) Anomalies of FWC in the historical simulation and observations (*7*, *8*, *35*) for the (A) Beaufort Gyre and (B) Eurasian Basin relative to the mean values over the shown observational period. The error bars show the uncertainties of observational estimates. (**C** and **D**) FWC in the historical and perturbation simulations for the (C) Beaufort Gyre and (D) Eurasian Basin.

**Fig. 3.**
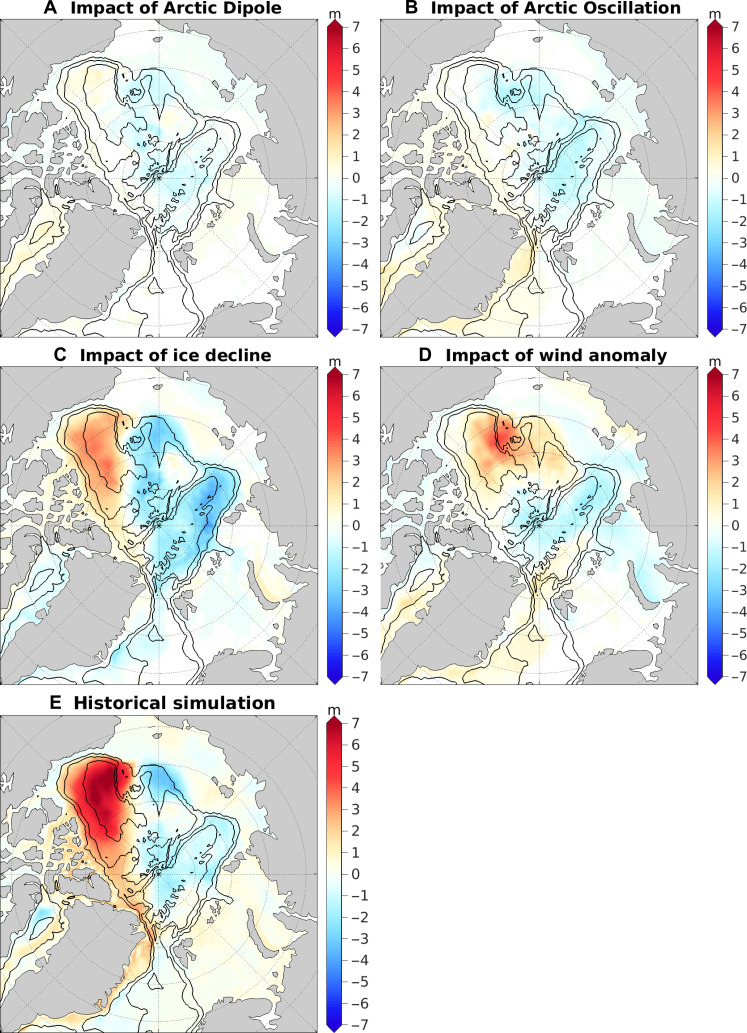
Impacts of winds and sea ice decline on the spatial patterns of liquid FWC. (**A** to **D**) Changes in FWC induced by (A) wind forcing of the Arctic Dipole, (B) wind forcing of the Arctic Oscillation, (C) sea ice decline, and (D) the full wind anomaly forcing averaged over 2018 to 2021. (**E**) Anomaly of FWC averaged over 2018 to 2021 relative to the climatological mean (1980 to 2000) in the historical simulation. The black contour lines indicate the 500-, 2000-, and 3500-m isobaths.

The Beaufort Gyre region accumulated a large amount of fresh water during the latter halves of the 2000s and 2010s (Fig. 2A) (*5*–*8*), reaching a level ~40% higher than the 1970s (*7*). In the Eurasian Basin, the freshwater content (FWC) increased at the beginning of the 2000s following the return of the Arctic Oscillation from a strongly positive phase to neutral (*32*, *33*) and subsequently decreased during the latter half of the 2010s (Fig. 2B). The spatial pattern of the FWC anomaly between the recent period (2018 to 2021) and the climatological mean (1980 to 2000) clearly indicates recent opposing changes in different basins: freshwater accumulation in the Beaufort Gyre and freshwater reduction, thus an increase in salinity, in the upper Eurasian Basin (Fig. 3E). The salinity changes in the upper Eurasian Basin in the 2010s are also evident in the depth-time plot of salinity (fig. S6B).

Eliminating either the total wind anomaly (corresponding to the SLP anomaly in Fig. 1A) or sea ice decline can markedly reduce the FWC in the Beaufort Gyre region (Fig. 2C). When comparing the FWC in the Beaufort Gyre region at the end of the simulations, simulations without sea ice decline or total wind anomaly forcing exhibit similar values, approximately half of the value in the historical simulation (Fig. 2C). Conversely, the influence of wind forcings associated with the Arctic Dipole and Arctic Oscillation changes (corresponding to the SLP anomalies in Fig. 1, C and F) on the Beaufort Gyre is comparatively minor. These wind forcings slightly decrease the FWC in the western Canada Basin (Fig. 3, A and B).

All considered factors contribute to a reduction in FWC in the Eurasian Basin, with sea ice decline exerting the most prominent influence (Figs. 2D and 3). In the absence of sea ice decline, the FWC in the Eurasian Basin in the 2010s surpasses that of the 2000s, indicating the important role of sea ice decline in reducing the FWC in this region. Both sea ice decline and the total wind anomaly forcing have a dipole imprint on Arctic freshwater distribution, with an increase in the Canada Basin and a reduction in the Eurasian Basin (Fig. 3, C and D). The reduction in the Eurasian Basin induced by sea ice decline roughly offsets the increase in the Beaufort Gyre region (Figs. 2, C and D, and 3C), while the total wind anomaly forcing contributes to an overall increase in Arctic FWC (Fig. 3D).

The reduction in the FWC in the Eurasian Basin due to altered winds is consistent with expectations based on cyclonic atmospheric circulation anomalies (Fig. 1, A, C, and F), which induce Ekman divergence and subsequent release of fresh water. Altering winds also affects sea ice thermodynamics by influencing sea ice dynamics, which can lead to changes in ocean surface freshwater flux (fig. S7). However, this impact on the Arctic freshwater budget is relatively minor compared to the influence of sea ice decline associated with thermal forcing (fig. S8A). Sea ice decline increases both ocean surface freshwater flux and ocean surface stress in the Arctic basin (fig. S8). Given the increased surface freshwater flux into the ocean in the Eurasian Basin (fig. S8A), the reduction in FWC in this region under the condition of sea ice decline (Fig. 3C) is induced by increased ocean surface stress (fig. S8B). Climatological winds in the Arctic render surface Ekman transport directed from the Eurasian Basin to the Amerasian Basin as a result of higher SLP over the Canada Basin (the atmospheric Beaufort High) compared to the Eurasian Basin (*34*). Sea ice decline intensifies ocean surface stress and thus the Ekman transport. This change, in turn, reduces the FWC in the Eurasian Basin while increasing it in the Amerasian Basin (*35*, *36*).

Our comparison reveals the predominance of sea ice decline in driving changes in FWC in the Eurasian Basin, overshadowing the impacts of individual wind forcing (Figs. 2D and 3, A to D). The relatively small effects of the Arctic Dipole anomaly can be attributed in part to its limited magnitude (Fig. 1C). The Arctic Oscillation forcing and the total wind anomaly forcing have slightly larger impacts than the Arctic Dipole forcing (Fig. 2D).

### Warming of the Arctic Ocean

**Fig. 4.**
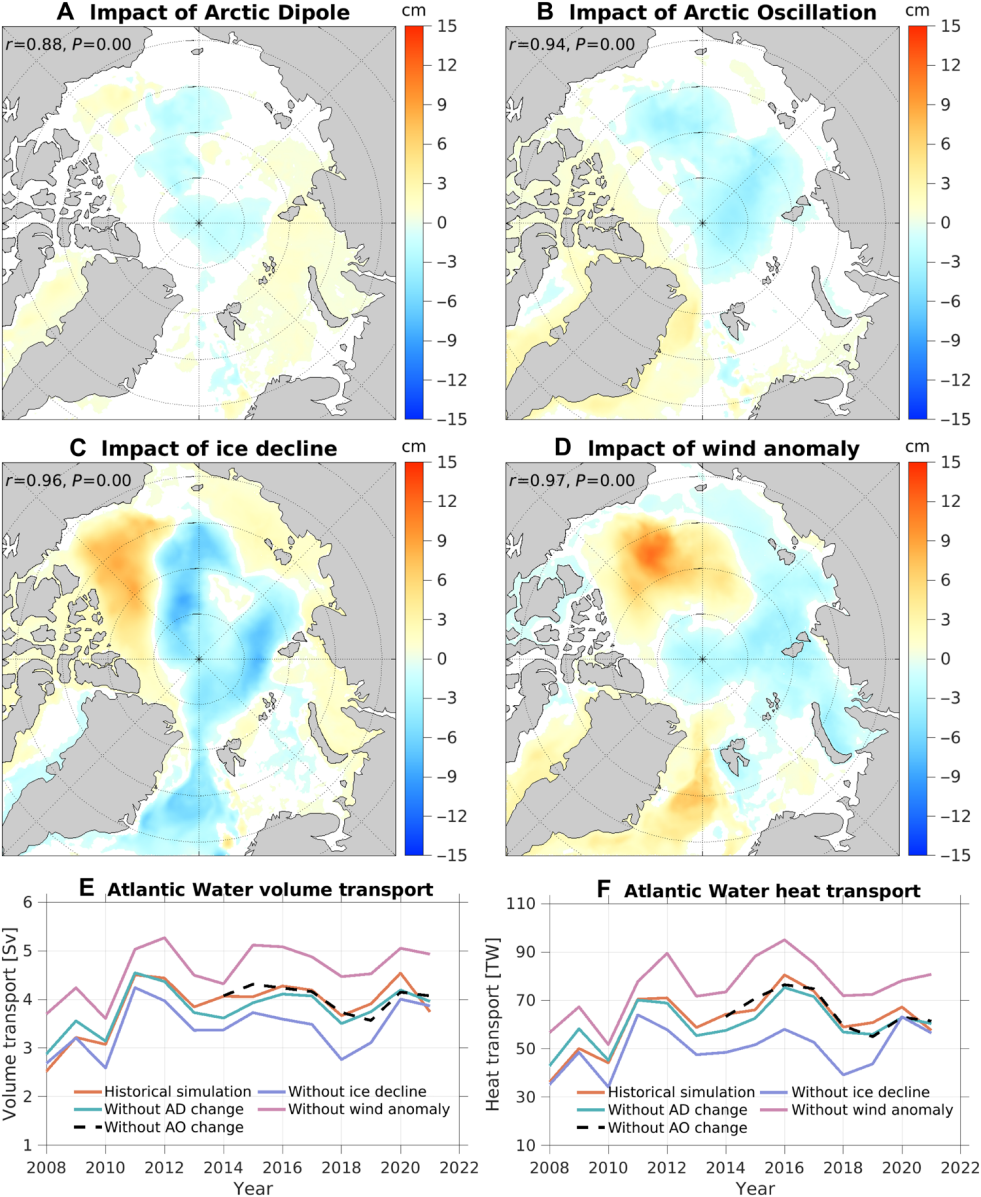
Impacts of winds and sea ice decline on the SSH and Fram Strait inflow. (**A** to **D**) Changes in SSH induced by (A) wind forcing of the Arctic Dipole, (B) wind forcing of the Arctic Oscillation, (C) sea ice decline, and (D) the full wind anomaly forcing averaged over 2018 to 2021. The spatial correlation coefficients between the SSH anomalies and the corresponding FWC anomalies (shown in Fig. 3) are indicated in each panel. (**E** and **F**) Atlantic Water (E) volume and (F) heat transports through the Fram Strait in the historical and sensitivity simulations.

The sea surface height (SSH) anomalies between the historical simulation and the sensitivity simulations are primarily due to halosteric height changes, hence exhibiting high spatial correlation with FWC anomalies in the Arctic Ocean (compare Fig. 3, A to D, and Fig. 4, A to D). Cyclonic winds induce Ekman divergence and a decrease in SSH, whereas anticyclonic winds lead to Ekman convergence and an increase in SSH (*37*, *38*). Therefore, wind forcings associated with the Arctic Dipole and Arctic Oscillation anomalies (Fig. 1, C and F) result in negative SSH anomalies in the Eurasian Basin (Fig. 4, A and B). The total wind anomaly forcing, characterized by a tripole pattern (Fig. 1A), produces a corresponding tripole pattern in the SSH anomaly (Fig. 4D).

Sea ice decline triggers a pronounced SSH reduction spanning the Eurasian and Makarov basins and an increase in SSH in the eastern Canada Basin (Fig. 4C). In addition, sea ice decline induces a negative SSH anomaly in the Greenland Sea (Fig. 4C), attributed to a positive salinity anomaly resulting from reduced sea ice freshwater export from the Arctic Ocean into the Greenland Sea (*20*). The SSH anomalies in the Greenland Sea in the cases of sea ice decline and total wind anomaly forcing are not reflected in changes in FWC as most of the water in the Greenland Sea has salinity higher than the upper threshold used to define the FWC.

A strengthening of the cyclonic Greenland Sea gyre is known to drive warm Atlantic Water toward the Fram Strait, thereby increasing its inflow into the Arctic Ocean (*17*–*19*, *21*). Similarly, an intensification of the cyclonic circulation in the Eurasian Basin has an effect to draw in Atlantic Water through the Fram Strait. On the contrary, anticyclonic anomalies of ocean circulation in these basins tend to reduce the Atlantic Water inflow through the Fram Strait. The decline of sea ice leads to negative SSH anomalies and thus cyclonic circulation anomalies in both the basins (Fig. 4C), which consequently amplifies volume and heat transports into the Arctic Ocean through the Fram Strait (Fig. 4, E and F).

The full wind anomaly forcing leads to a positive SSH anomaly and thus an anticyclonic circulation anomaly in the Greenland Sea, which extends into the western Eurasian Basin (Fig. 4D). This results in a reduction in volume and heat transports through the Fram Strait (Fig. 4, E and F). On the contrary, wind forcings associated with the Arctic Dipole and Arctic Oscillation lead to statistically insignificant increases in Atlantic Water inflow in the later periods of the corresponding simulations (Fig. 4, E and F) in response to the cyclonic circulation anomalies in the Eurasian Basin (Fig. 4, A and B). It is worth noting that the volume and heat transports through the Barents Sea Opening remain largely unaffected by the considered forcing perturbations (fig. S9).

**Fig. 5.**
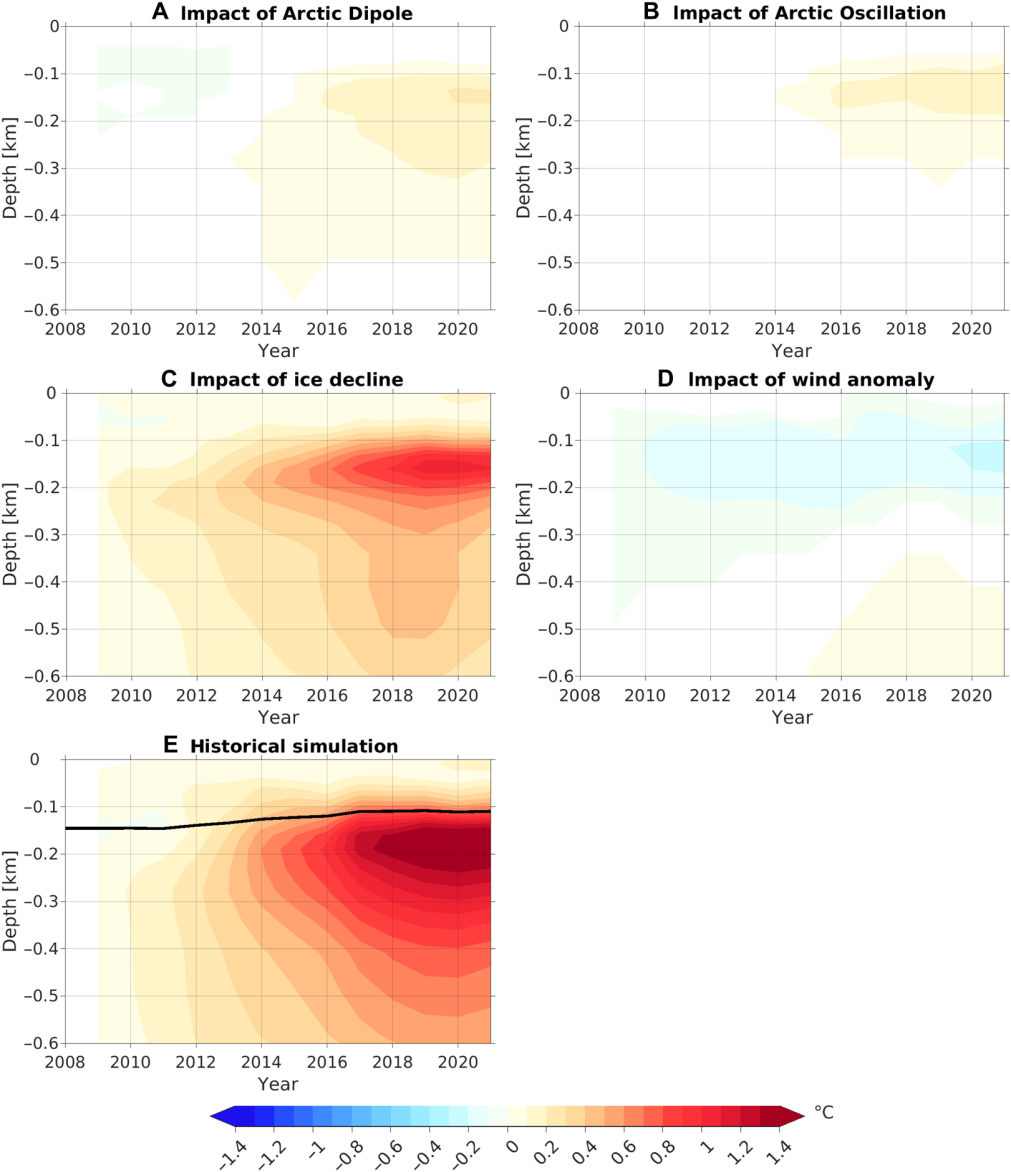
Depth-time plots of temperature anomalies relative to 2008 in the Eurasian Basin. (**A** to **D**) Changes in temperature induced by (A) wind forcing of the Arctic Dipole, (B) wind forcing of the Arctic Oscillation, (C) sea ice decline, and (D) the full wind anomaly forcing. (**E**) Temperature anomaly in the Eurasian Basin in the historical simulation. The black contour denotes the 0°C isotherm.

**Fig. 6.**
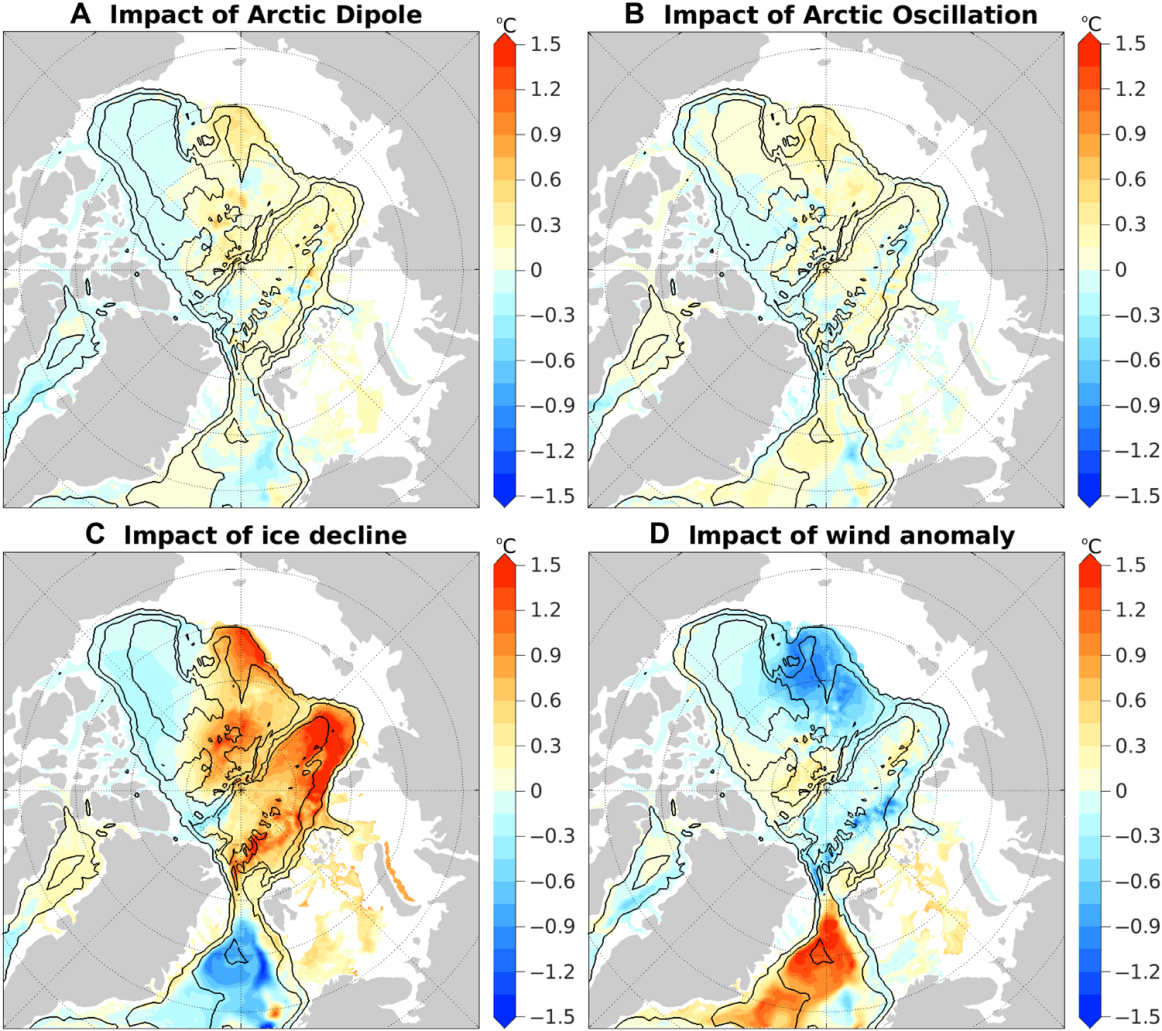
Temperature changes at 250-m depth induced by forcing perturbations. (**A** to **D**) Temperature changes induced by (A) wind forcing of the Arctic Dipole, (B) wind forcing of the Arctic Oscillation, (C) sea ice decline, and (D) the full wind anomaly forcing averaged over the last four model years of the simulations. The black contour lines indicate the 500-, 2000-, and 3500-m isobaths.

Consistent with their impacts on the Fram Strait inflow, both the Arctic Dipole and Arctic Oscillation contribute to the warming of the Atlantic Water layer in the Eurasian Basin during the latter half of the 2010s (Figs. 5, A and B, and 6, A and B). However, their effects are much smaller than the impacts of sea ice decline (Figs. 5C and 6C). In the case of sea ice decline, the increase in warm Atlantic Water inflow to the Arctic basin through the Fram Strait (Fig. 4, E and F) results in a reduction in the recirculation of Atlantic Water from the Fram Strait to the Greenland Sea, inducing a cold anomaly in the Greenland Sea (Fig. 6C). The cyclonic circulation anomaly that spans both the Eurasian and Makarov basins (Fig. 4C) facilitates the warm anomaly to propagate into the Makarov basin (Fig. 6C).

The full wind anomaly forcing, specifically the high SLP anomaly over the Greenland Sea, diminishes the Atlantic Water inflow in the Fram Strait (Fig. 4, E and F), thus producing an effect opposite to that of sea ice decline, characterized by a warm anomaly in the Greenland Sea and a decrease in temperature in the Arctic basin (Fig. 6D). In this case, the strengthening of the surface anticyclonic circulation in the western Canada Basin and Makarov Basin (Fig. 4D) acts to weaken the cyclonic circulation of the Atlantic Water layer, consequently intensifying the cold anomaly in the western Canada Basin and Makarov Basin while alleviating the cold anomaly in the Eurasian Basin (Figs. 5D and 6D).

Overall, sea ice decline is the key factor driving the warming and uplift of the Atlantic Water layer (Figs. 5 and 6). It alone contributes ~50% to the increase in ocean heat content in the upper 600 m of the Eurasian Basin during the 2010s. Other factors, including the long-term warming trend in the inflow water (fig. S10), which represents a signal of climate warming (*21*), and the overall effects of wind changes, collectively explain the remaining half of the warming.

## DISCUSSION

Located near the Arctic gateways of Atlantic Water inflows, the southwestern Barents Sea has been experiencing a warming trend that dates back at least to the 1980s (*15*, *21*). This process of atlantification in the southwestern Barents Sea, where sea ice remains absent year-round, is driven by the warming of Atlantic Water inflow through the Barents Sea Opening. The concurrent atmospheric warming in this region reduces the heat loss from the ocean, further amplifying the ocean warming (*25*, *26*). This increase in ocean heat content leads to the retreat of sea ice, enhanced surface heat loss, and weakened stratification downstream in the Barents Sea, contributing to a poleward expansion of Arctic atlantification over the continental shelf (*27*, *39*, *40*). The long-term ocean warming trend is accompanied by warming and cooling events. On interannual and shorter timescales, local winds can strongly modulate the Atlantic Water heat transport and sea ice cover in the Barents Sea (*41*, *42*). In addition, winter atmospheric cyclones in the Barents Sea can enhance ocean mixing, facilitating the transfer of Atlantic Water heat to the subsurface and thereby strengthening atlantification (*43*).

In the Eurasian Basin, regional weather can also influence upper ocean mixing and vertical ocean heat flux on short timescales (*44*, *45*), similar to observations in the Barents Sea. Furthermore, the weakening of the halocline and the warming of the Atlantic Water layer in the Eurasian Basin could potentially trigger air-ice-sea feedback mechanisms, akin to those in the Barents Sea, thereby promoting regional sea ice loss (*13*, *14*). Now, the region experiencing the most intense Atlantic Water heat loss and upper ocean mixing is located in the Barents Sea; however, this region is shifting toward the Arctic interior as climate warming continues (*27*, *46*). It is crucial to understand the poleward expansion of Arctic atlantification along both branches of Atlantic Water inflows under a warming climate as different driving processes could be at play. In this study, we focused on understanding the main drivers of Arctic atlantification in the Eurasian Basin in the 2010s, with particular emphasis on the roles of large-scale sea ice decline and wind forcing on multiyear to decadal timescales.

Recent changes in Arctic atmospheric circulation have revealed a tripole pattern, characterized by anticyclonic circulation anomalies over the Canada Basin and Greenland Sea, alongside a cyclonic circulation anomaly over the Barents-Kara seas (Fig. 1A). The intensification of atmospheric cyclone activity in the Arctic, a trend correlated with a warming climate (*47*), has emerged as an important factor influencing the Beaufort High situated over the Canada Basin (*48*, *49*). Cyclones originating from the North Atlantic could decrease the SLP along their trajectories before reaching the Canada Basin, thereby reinforcing anticyclonic winds around the Canada Basin (as depicted by negative wind curl in Fig. 1L) and consequently augmenting the FWC of the Beaufort Gyre during the 2010s (*30*). The decadal variability in cyclone frequency, which leaves its mark on the SLP, has been recognized for its potential to influence the inflow of Atlantic Water into the Arctic Ocean (*42*). The cyclone frequency over the Greenland Sea experienced a decline during the 2010s (*50*), aligning with the positive SLP anomaly observed in the region (Fig. 1A). Thus, both the quantity of cyclones originating from lower latitudes and their trajectories—whether traversing the Greenland Sea or not—can affect the strength of the Greenland Sea gyre and the inflow in the Fram Strait.

The combined effects of poleward atmospheric moisture transport, poleward oceanic heat transport, sea ice retreat, and different climate feedback processes have substantially amplified the atmosphere warming in the Barents-Kara seas (*2*, *26*, *51*–*53*). The amplified atmosphere warming, in turn, led to a reduction in SLP in the region. Therefore, the recent tripole changes in Arctic atmospheric circulation likely resulted from a combination of natural variability and the influences of climate change. Notably, our analysis indicates that these atmospheric changes do not conform to the decadal alternation of the atmospheric Arctic Dipole, in contrast to prior assertions (*23*).

Among the three poles of the SLP anomalies (Fig. 1A), the two located inside the Arctic polar region contributed to an increase in FWC in the Beaufort Gyre while reducing it in the Eurasian Basin. It is important to note that this dipole SLP pattern should not be confused with the positive-phase Arctic Dipole pattern. On the one hand, the decadal variability of the Arctic Dipole cannot account for the recent SLP changes (Fig. 1, A and C). On the other hand, the exact positioning of the positive SLP anomaly center holds significance for freshwater accumulation. Despite their spatial proximity, a positive SLP anomaly centered over the Beaufort Gyre region is capable of accumulating substantially more fresh water than an anomaly centered over the northern Canadian Arctic Archipelago (see fig. S11). Moreover, the positive SLP anomaly over the Greenland Sea, which bears no resemblance to the pattern of the Arctic Dipole, acted to reduce the inflow of warm Atlantic Water through the Fram Strait and induced a cooling effect on the Arctic basin. In contrast, the recent decadal variability in the Arctic Dipole led to a slight increase in temperature in the Arctic basin by generating a weak cyclonic circulation anomaly in the Eurasian Basin. As a result, the Arctic Dipole did not weaken the recent Arctic atlantification, contrary to the previous suggestion (*23*).

We found that sea ice decline exerted the most prominent influence on the spatial distribution of Arctic fresh water and the warming of the Atlantic Water layer, acting as the primary driver of atlantification in the Arctic deep basin. It engendered a negative FWC anomaly in the Eurasian and Makarov basins, leading to a cyclonic circulation anomaly, which facilitated the warm anomaly in the Atlantic Water layer to propagate through the Makarov Basin. This effect is similar to that of a positive phase of the Arctic Oscillation (*54*). During the 1990s, the notably positive Arctic Oscillation (Fig. 1I) strengthened the cyclonic circulation in the Arctic Ocean (*55*–*57*), while concurrently, the positive phase of the North Atlantic Oscillation increased the Atlantic Water inflow through the Fram Strait (*58*). During the 2010s, the positive Arctic Oscillation also tended to reinforce the cyclonic ocean circulation; however, its relatively weak positive phase exerted a weaker effect on Arctic FWC and ocean circulation. Instead, sea ice decline emerged as the primary driver, yielding an even greater impact on diminishing FWC in the Eurasian and Makarov basins and augmenting the Atlantic Water inflow as well. Consequently, recent changes in the Arctic Ocean are predominantly shaped by the influence of climate warming, different from the natural variability-induced changes in the 1990s. The emergence of Arctic atlantification in the Arctic deep basin during the 2010s, primarily due to climate warming, indicates a transition to a distinct climate state.

The influence of sea ice decline on Arctic Ocean heat content suggests a feedback loop, wherein ocean heat could increase sea ice basal melting and atmospheric warming. This process could be particularly important in the context of continued ocean warming in future climate scenarios (*46*, *53*). The present study used forced ice-ocean model simulations with forcing perturbations to isolate and identify the individual contributions of different factors to the emergence of atlantification in the Arctic deep basin. By using prescribed atmospheric forcing, we were able to conduct an attribution analysis along the actual climate trajectory experienced. To better understand possible feedback mechanisms between the ocean, sea ice, and atmosphere during the progression of Arctic atlantification, high-resolution coupled climate models are needed in future studies.

## MATERIALS AND METHODS

### Terminology

In this study, the Arctic Ocean refers to the Arctic Basin (Eurasian and Amerasian basins) and its surrounding shelf seas (see fig. S1).

### Calculation of SLP anomaly

We performed an Empirical Orthogonal Function (EOF) analysis on deseasoned monthly SLP data north of 70°N, following the methodology used in a previous study (*59*). We used SLP data spanning the period of 1960 to 2021 from the atmospheric reanalysis JRA55-do (*60*). The first three leading modes (patterns depicted in fig. S3) explain 66% (Arctic Oscillation), 12% (Arctic Dipole), and 9% of the total variance, respectively.

By multiplying the leading modes with their corresponding principal components (time series depicted in Fig. 1, I to K), we reconstructed the time series of SLP changes associated with these modes. For example, the time series of SLP associated with the first mode can be calculated as follows

$${\text{SLP}}_{1}\left(x,y,t\right)={\text{EOF}}_{1}\left(x,y\right)\cdot {\text{PC}}_{1}\left(t\right)$$ (1)

Here, EOF_1_ represents the first mode, and PC_1_ is the corresponding principal component. Then, we can calculate the temporal mean of SLP_1_ for different time periods and evaluate the differences between these periods.

Following previous research concerning the impact of the Arctic Dipole on Arctic atlantification (*23*), we calculated the SLP difference associated with the Arctic Dipole between the periods of 2007 to 2021 and 1992 to 2006 (Fig. 1C). Similarly, we calculated the SLP difference linked to the Arctic Oscillation between the period of 2014 to 2021 and the long-term mean over 1960 to 2021 (Fig. 1F). The Arctic Oscillation was mainly in a positive phase during the period of 2014 to 2021 (Fig. 1I).

### Model simulations

We used the global Finite-Element Sea-Ice Ocean Model (FESOM) (*61*, *62*) in this study. It works on unstructured variable-resolution meshes. Our model configuration featured a high horizontal resolution of 4.5 km in the Arctic Ocean and relatively coarser resolution elsewhere. The vertical grid spacing was set to 10 m in the upper 100 m and gradually coarsened with depth. We initialized the historical simulation from the PHC3 hydrography climatology (*63*) and forced the model with the JRA55-do atmosphere reanalysis fields and runoff (*60*) for the period from 1958 to 2021. The model can reasonably simulate the warming trends of Atlantic Water inflow in the Fram Strait (fig. S10) and the declining trends of Arctic sea ice (fig. S2, A and B). The warming and saline trends in the Eurasian Basin during the 2010s are consistently simulated in FESOM and the high-resolution Ocean Model Intercomparison Projection (OMIP) models (fig. S6) (*64*). Although the FESOM simulation overestimates the mean value of FWC in the Beaufort Gyre by 11%, it captures the observed temporal variations over the past two decades (Fig. 2A).

To disentangle the impacts of different factors on the process of atlantification, we carried out four sensitivity simulations, comprising three wind-perturbation simulations and one thermal forcing simulation. In the wind-perturbation simulations, we subtracted from wind forcing the wind anomalies suggested to be important contributors to recent changes in the Arctic Ocean. The first case is intended to investigate the impact of the decadal alternation in the Arctic Dipole in the period of 2007 to 2021, relative to the preceding period of 1992 to 2006, which was proposed to have substantially mitigated recent atlantification (*23*). For this experiment, we derived near-surface wind corresponding to the SLP anomaly associated with the changes in the Arctic Dipole between these two periods (as depicted in Fig. 1C). This wind-perturbation simulation was started from the historical simulation results at the beginning of 2007 and performed till 2021. It differed from the historical simulation only in the subtraction of the wind anomaly from the wind forcing.

The other wind-perturbation simulations are different from the first one in terms of the subtracted wind anomalies. In the second wind-perturbation simulation, we subtracted the wind corresponding to the SLP anomaly associated with the positive phase of the Arctic Oscillation during the period of 2014 to 2021 (Fig. 1F). The shift of the Arctic Oscillation to its positive phase since 2014 has been suggested to enhance the cyclonic circulation mode of the Arctic Ocean (*54*) and possibly contribute to freshwater accumulation in the Canada Basin (*29*). In the last wind-perturbation simulation, we subtracted the wind corresponding to the entire SLP anomaly between 2007 to 2021 and 1992 to 2006 (Fig. 1A), which shows a tripole pattern and does not align with the variability of the Arctic Oscillation and Arctic Dipole.

The simulation using perturbed thermal forcing spanned the period of 2001 to 2021 and is intended to eliminate the impact of Arctic sea ice decline in this period. In this simulation, we substituted the thermal forcing fields, including near-surface air temperature and downward shortwave and longwave radiation fluxes inside the Arctic region, with their climatological values. The climatological values were derived by averaging the JRA55-do forcing fields from 1970 to 1999 for each forcing record (3 hourly). It was suggested that using such climatological thermal forcing can effectively eliminate the declining trend of Arctic sea ice in model simulations (*35*), as depicted in fig. S2. Aside from the replacement of thermal forcing, this simulation maintained the same configuration as the historical simulation, including wind forcing. In all the simulations, surface turbulent fluxes over ice and ocean are calculated using bulk formulas. This approach ensures that the impacts of sea ice decline and wind anomalies on the ocean are represented in the simulations through changes in ocean surface stress and freshwater flux. It is also worth noting that, in all simulations, the sea ice module simulates both dynamic and thermodynamic changes, with the sea ice state not being manually specified.

The difference in the model results between the historical simulation and sensitivity simulations reveals the impact of the modified forcing. It is worth noting that the sensitivity simulations cover distinct time periods, and it is conceivable that the impacts of the considered factors on the ocean may exhibit interdependencies. However, our primary objective is to isolate and elucidate the individual impact of each considered factor. By systematically analyzing the differences between the historical simulation and the sensitivity simulations, we aim to disentangle the specific contributions of these factors to the observed changes in the Arctic Ocean.

### Impact of spatial positioning of SLP anomalies

The active center of the positive SLP anomaly during a positive Arctic Dipole phase is situated at the northern Canadian Arctic Archipelago (fig. S3B), distinguishing it from the location of the Beaufort High. To access the influence of the spatial positioning of positive SLP anomalies on freshwater accumulation, we further conducted two idealized wind-perturbation experiments.

In these experiments, the winds corresponding to the SLP anomalies depicted in fig. S11 (A and B) were respectively added to the wind forcing. One simulation represents an intensified Beaufort High, while the other represents the Arctic Dipole positive phase. Although the magnitudes of the positive SLP anomalies in both cases are the same, the induced changes in FWC are different, with the Arctic Dipole exerting a much smaller impact (fig. S11, C and D).

### Derivation of wind anomalies

We first calculated geostrophic wind (*u*_geo_, *v*_geo_) for each case of SLP anomalies and then used the following relation to derive near-surface wind velocities (*u*, *v*)

$$\left[\begin{array}{c} u\\ v \end{array}\right]=0.7\times \left[\begin{array}{cc} \text{cos}\left(30{}^{\circ}\right) & -\text{sin}\left(30{}^{\circ}\right)\\ \text{sin}\left(30{}^{\circ}\right) & \text{cos}\left(30{}^{\circ}\right) \end{array}\right]\left[\begin{array}{c} u_{\text{geo}}\\ v_{\text{geo}} \end{array}\right]$$ (2)

The scaling and deflection took into account the effect of surface friction on near-surface winds (*65*).

### Ocean volume and heat transports

We calculated the volume (OVT) and heat (OHT) transports of warm Atlantic Water through the Fram Strait and Barents Sea Opening as follows

OVT(t)=∬v(t)ds (3)

$$\text{OHT}\left(t\right)=\rho_{o}c_{p}\iint v\left(t\right)\left[T\left(t\right)-T_{\text{ref}}\right]ds$$ (4)

where ρ_o_ represents the ocean density, *c_p_* is the specific heat capacity of seawater, *v* is the ocean velocity perpendicular to the transect, *T* is the potential temperature, *T*_ref_ is the reference temperature set to 0°C, and the integration is performed over the vertical transect area where warm Atlantic Water is present. The definition of warm Atlantic Water in the Barents Sea Opening (>3°C) and Fram Strait (>2°C) follows previous studies (*66*, *67*).

### Freshwater content

The FWC in a water column quantifies the amount of pure water that is required to be removed from the column so that the mean salinity can be changed to the reference salinity. It is defined as follows

$$\text{FWC}=\int_{H}^{0}\left(S_{\text{ref}}-S\right)/S_{\text{ref}}dz$$ (5)

where *S* represents the salinity, *S*_ref_ denotes the reference salinity, and *H* is the depth at which the salinity equals the reference salinity. The reference salinity is set to 34.8 PSU (practical salinity unit), which is considered as the mean salinity of the Arctic Ocean (*68*). Estimates of Beaufort Gyre’s FWC based on observations have used the same reference salinity (*7*, *8*), allowing for direct comparisons with model simulations. The volumetric FWC in a region is obtained by integrating the FWC over this region.

## Supplementary Materials

### This PDF file includes:

Figs. S1 to S11 References
