# Supplementary material for: Recent emergence of Arctic atlantification dominated by climate warming
Source: Sci Adv. 2024 Nov 27;10(48):eadq5235. doi: 10.1126/sciadv.adq5235 (PMC11601200; doi:10.1126/sciadv.adq5235)
Supplement: Supplementary file 1 — Figs. S1 to S11 References [file sciadv.adq5235_sm.pdf]

Science Advances
AAAS

Supplementary Materials for

Recent emergence of Arctic atlantification dominated by climate warming

Qiang Wang *et al.*

Corresponding author: Qiang Wang, qiang.wang@awi.de; Fan Wang, fwang@qdio.ac.cn

Sci. Adv. **10**, eadq5235 (2024)
DOI: 10.1126/sciadv.adq5235

This PDF file includes:

Figs. S1 to S11
References

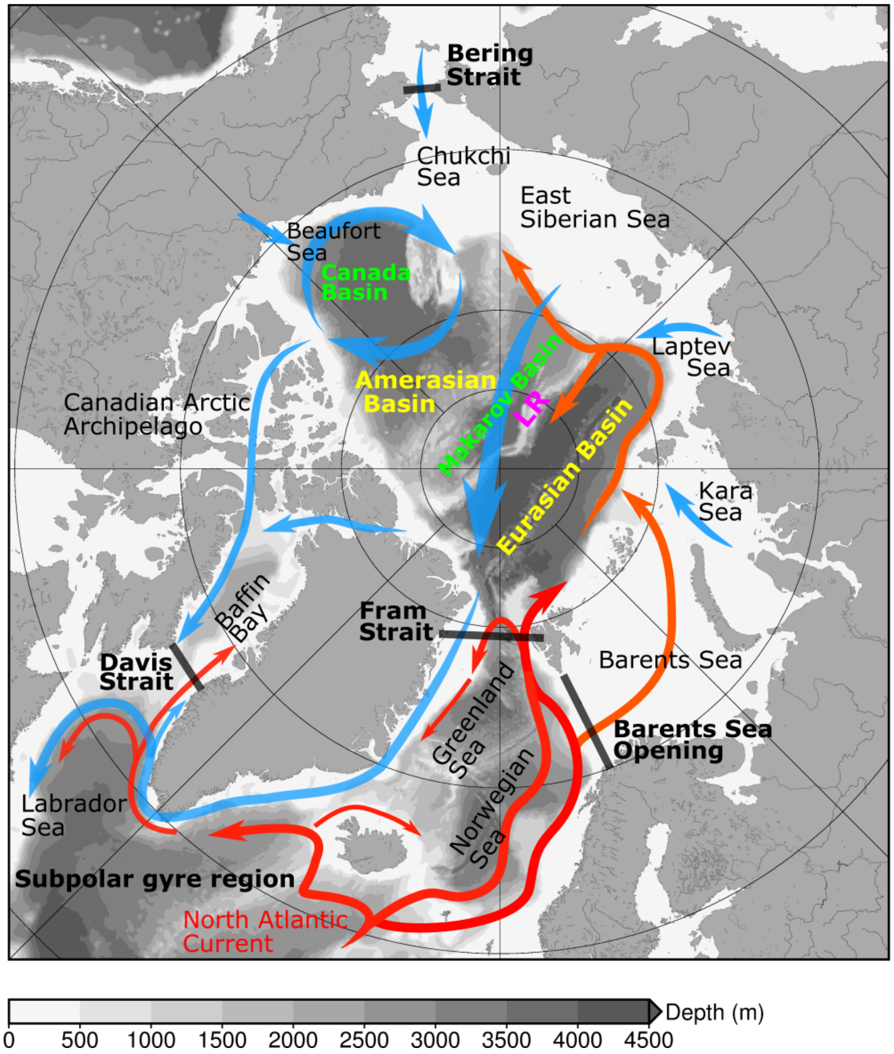

Fig. S1. Ocean bottom topography and schematic of ocean circulations in the pan-Arctic region (*21*). The blue and red arrows denote freshwater and Atlantic Water circulations, respectively. The Arctic Ocean consists of the Eurasian Basin, Amerasian Basin and the shelf seas in the region enclosed by Fram Strait, Davis Strait, Bering Strait and the Barents Sea Opening. The locations of the four Arctic gateways are indicated by thick black lines. LR stands for Lomonosov Ridge.

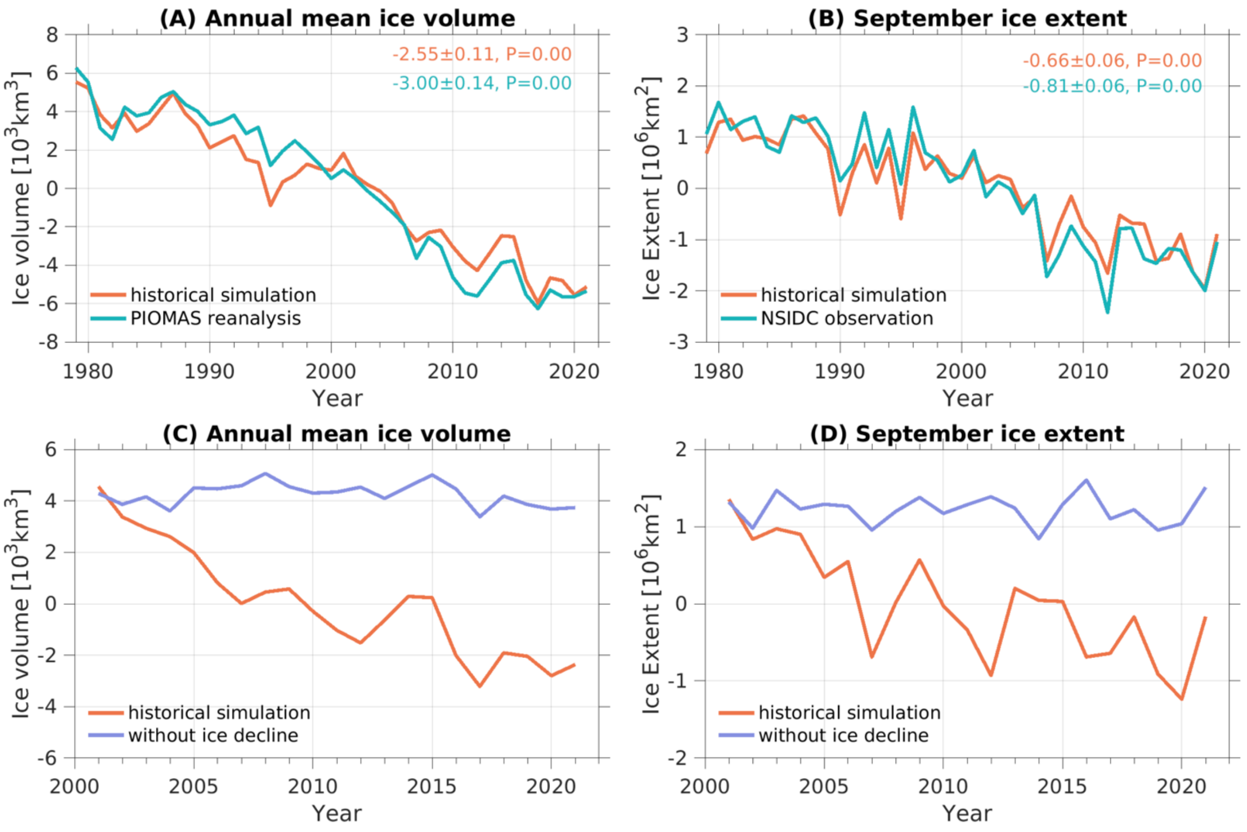

Fig. S2. Time series of Arctic sea ice volume and extent. (**A** and **B**) Time series of (A) annual sea ice volume and (B) September sea ice extent anomalies in the historical simulation, sea ice reanalysis (69) and satellite observations (*70*). (**C** and **D**) Anomalies of (C) sea ice volume and (D) sea ice extent in the historical simulation and the sensitivity simulation without sea ice decline; The anomalies are relative to the mean value of the historical simulation. The linear trends of Arctic sea ice volume (10^3km^3 per decade) and sea ice extent (10^6km^2 per decade) are indicated in (A) and (B), respectively.

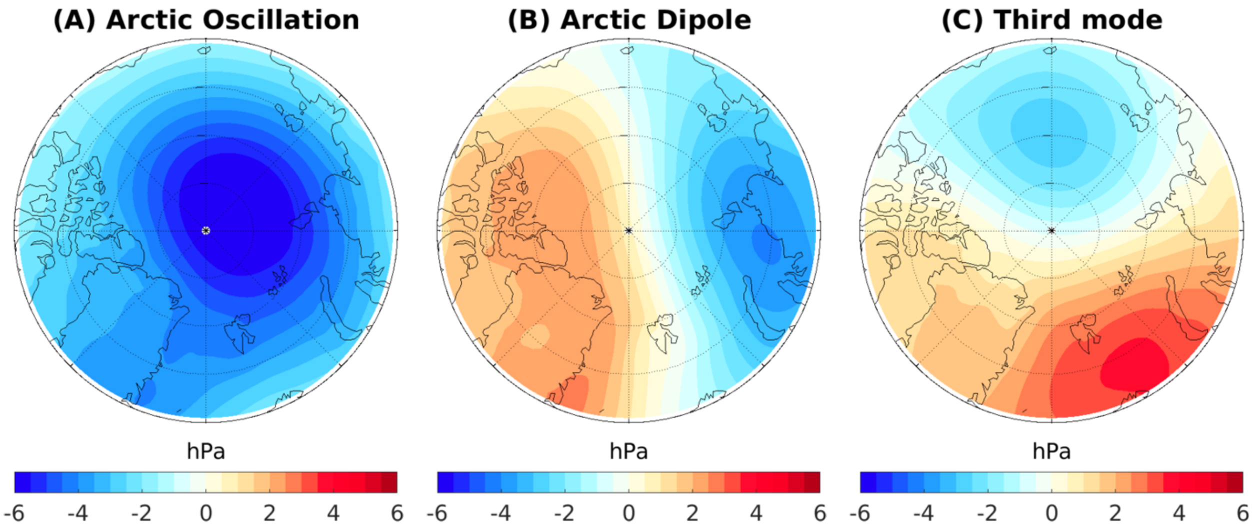

Fig. S3. Leading modes of Arctic atmospheric circulation. (A) The first mode: Arctic Oscillation. (**B**) The second mode: Arctic Dipole. (**C**) The third mode. Their corresponding principal components are shown in Fig. 1I-K.

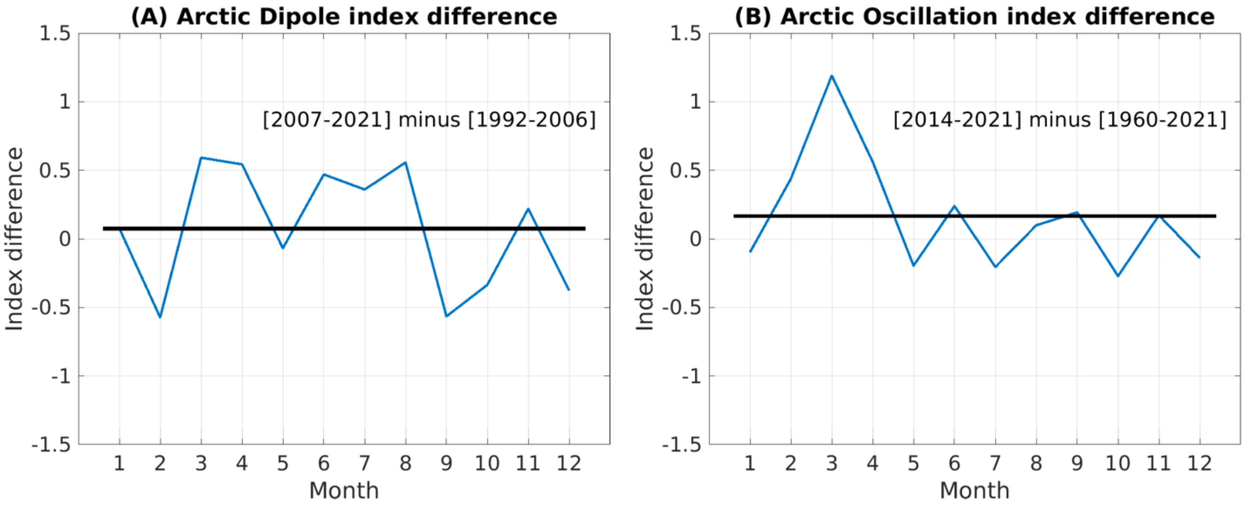

Fig. S4. Monthly anomalies of the Arctic Dipole and Arctic Oscillation indices. (**A**) The monthly difference of the Arctic Dipole index between 2007–2021 and 1992–2006. (**B**) The monthly difference of the Arctic Oscillation index between 2014–2021 and 1960–2021.

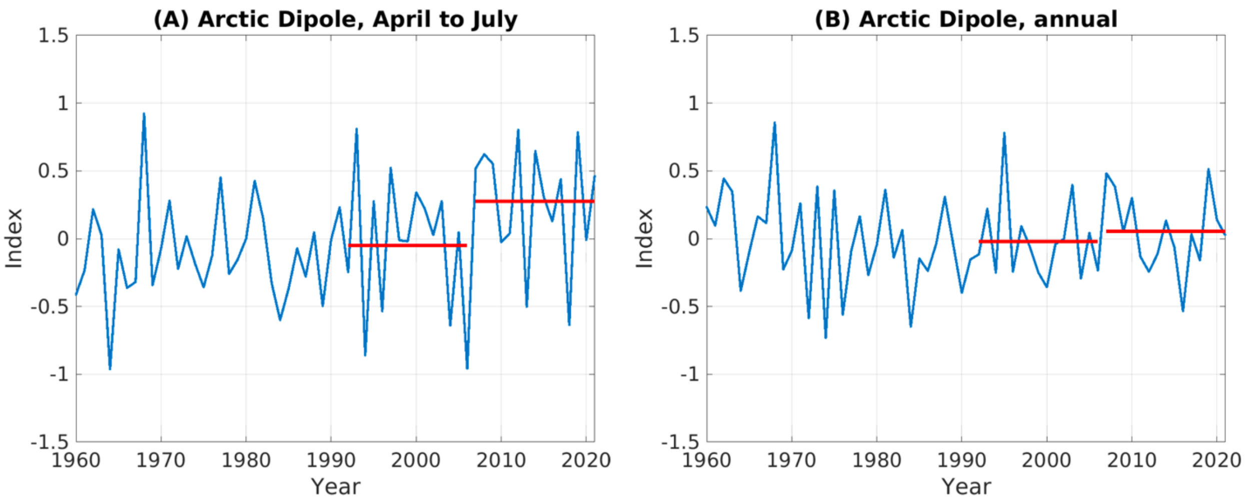

Fig. S5. Index of the Arctic Dipole. (**A**) The Arctic Dipole index for the season from April to July. (**B**) The same as (A), but for the annual mean index. The red lines denote the average over the two considered periods.

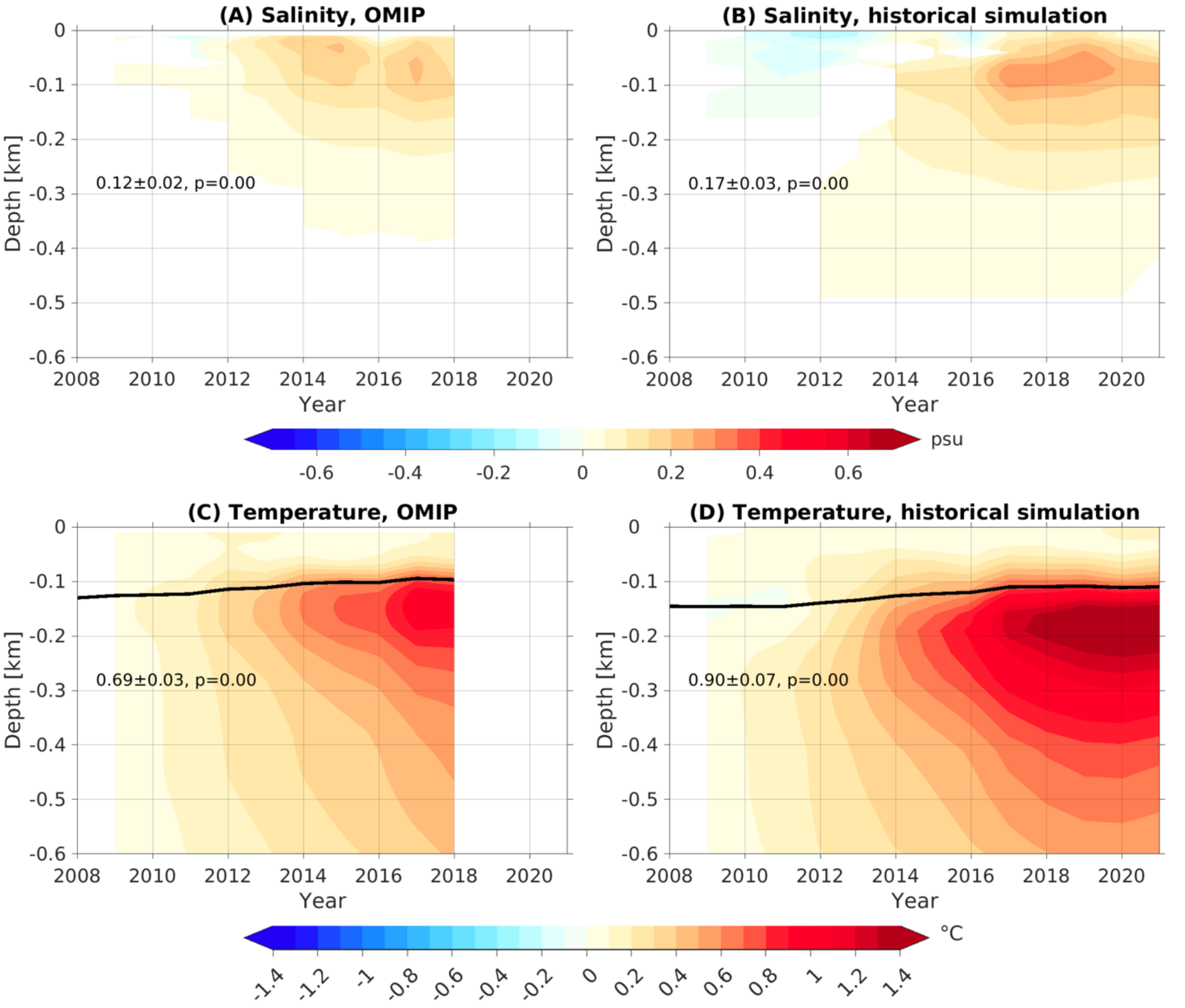

Fig. S6. Depth-time plots of salinity and temperature anomalies relative to 2008 in the Eurasian Basin. (**A** and **B**) Salinity anomalies in (A) Ocean Model Intercomparison Project (OMIP) simulations and (B) FESOM historical simulation. (**C** and **D**) Temperature anomalies in (C) OMIP simulations and (D) FESOM historical simulations. The black contours denote the 0°C isotherm. OMIP data are the multi-model-mean of four high-resolution OMIP models (ACCESS-MOM, AWI-FESOM, CMCC-NEMO and FSU-HYCOM) (*64*) and available for the period till 2018. The linear trends of salinity (psu per decade) and temperature (°C per decade) averaged over upper 300 m are indicated in each panel.

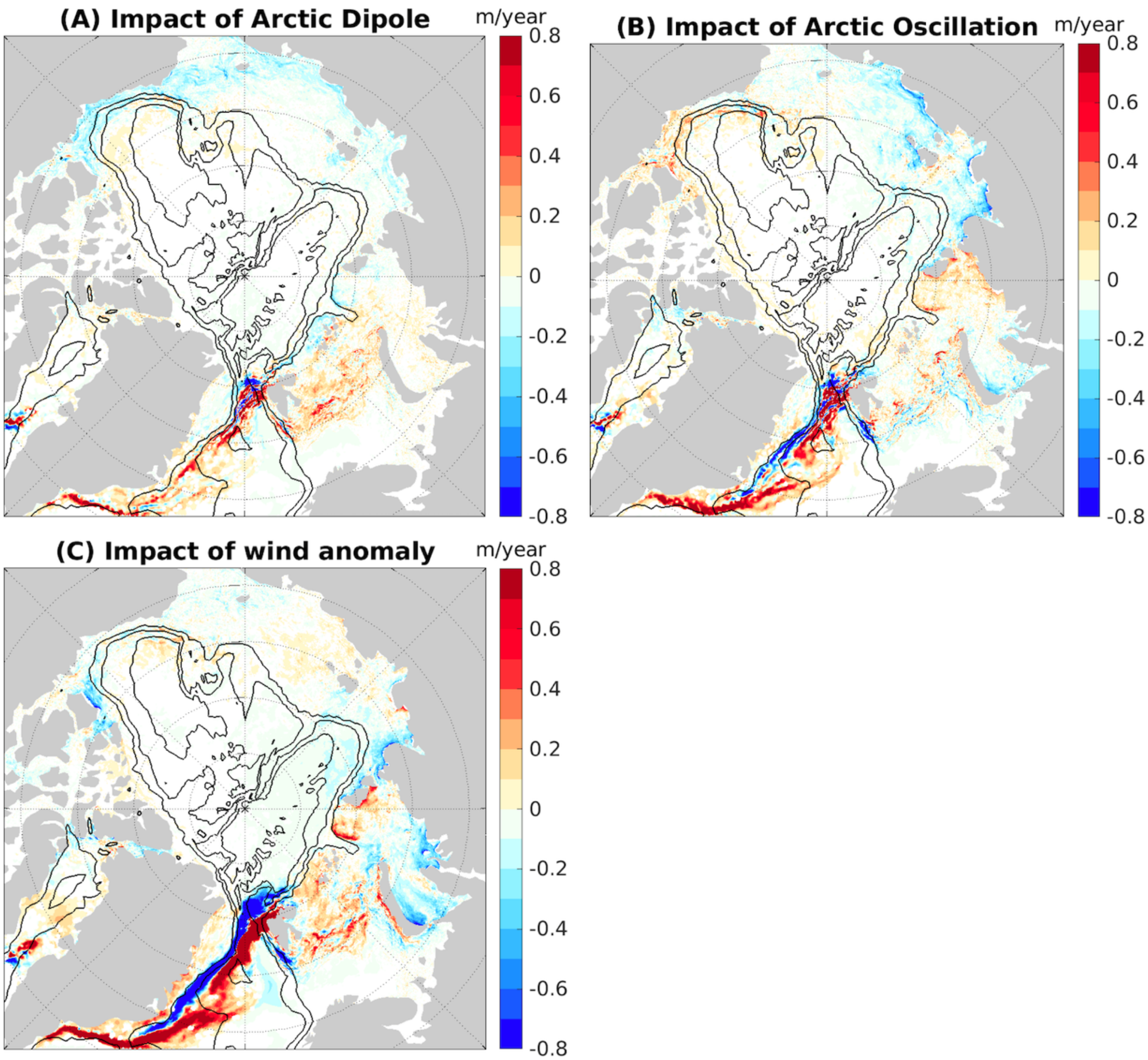

Fig. S7. Changes in ocean surface freshwater flux induced by wind perturbations. Changes induced by wind forcing of the (A) Arctic Dipole, (B) Arctic Oscillation, and (C) full wind anomaly. Fluxes into the ocean are indicated by positive values.

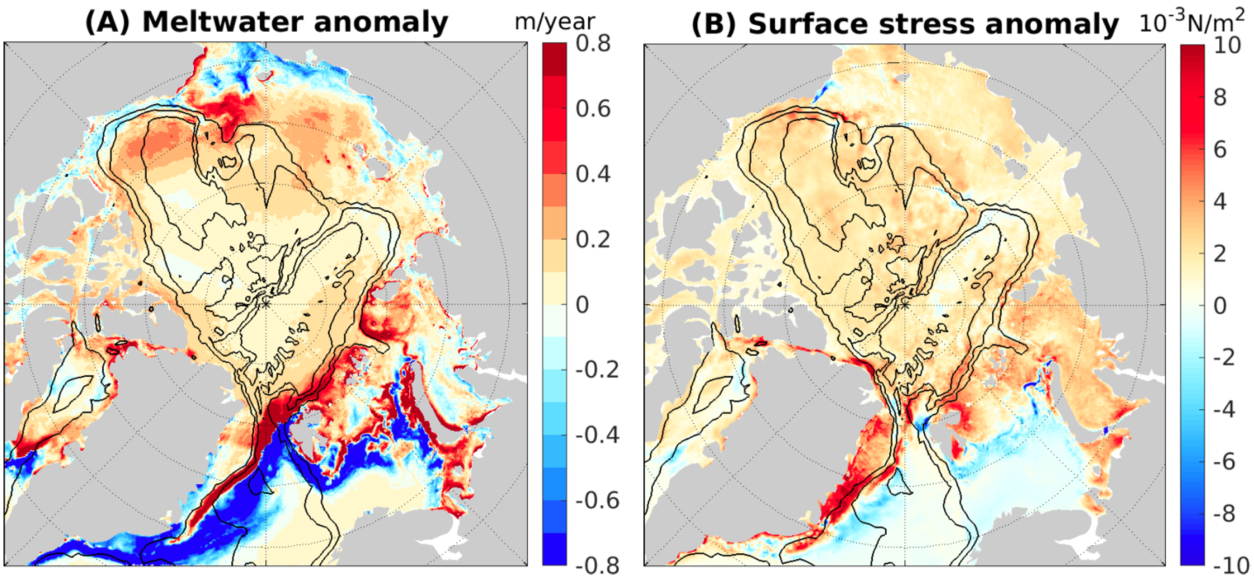

Fig. S8. Changes in ocean surface freshwater flux and stress induced by sea ice decline. (**A**) Changes in ocean surface freshwater flux associated with sea ice thermodynamics induced by sea ice decline. Freshwater fluxes into the ocean are positive. (**B**) Changes of ocean surface stress induced by sea ice decline.

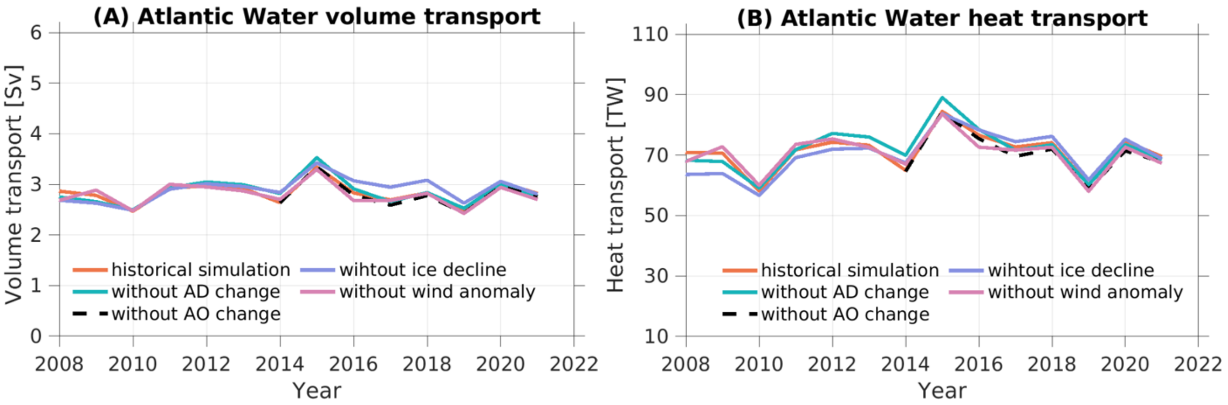

Fig. S9. Volume and heat transports of warm Atlantic Water in the Barents Sea Opening. Atlantic Water (A) volume and (B) heat transports in the historical control simulation and four sensitivity simulations.

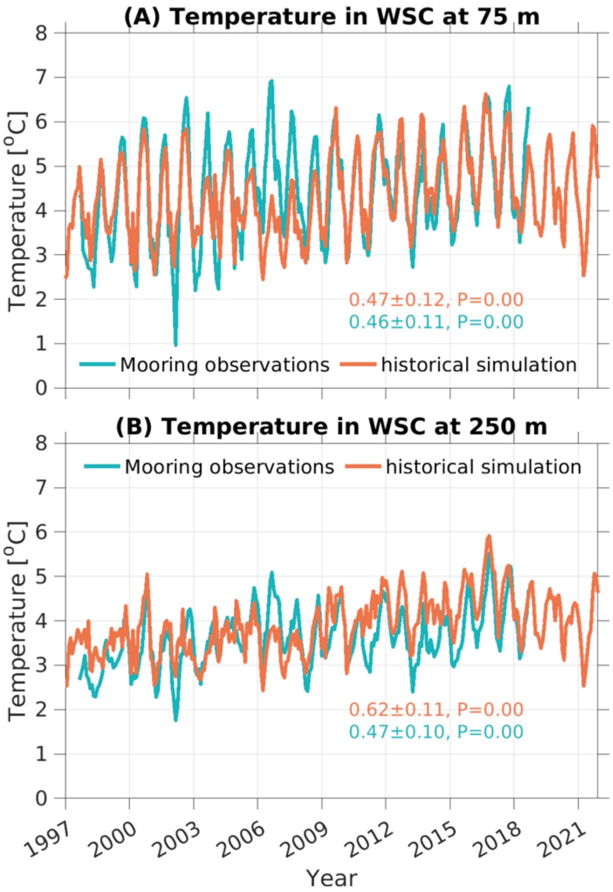

Fig. S10. Time series of temperature in the West Spitsbergen Current (WSC) in Fram Strait. Atlantic Water temperature in the WSC in the historical simulation and mooring observations (*71*): (A) at 75 m depth and (B) at 250 m depth. The linear trends of temperature (°C per decade) are indicated in each panel.

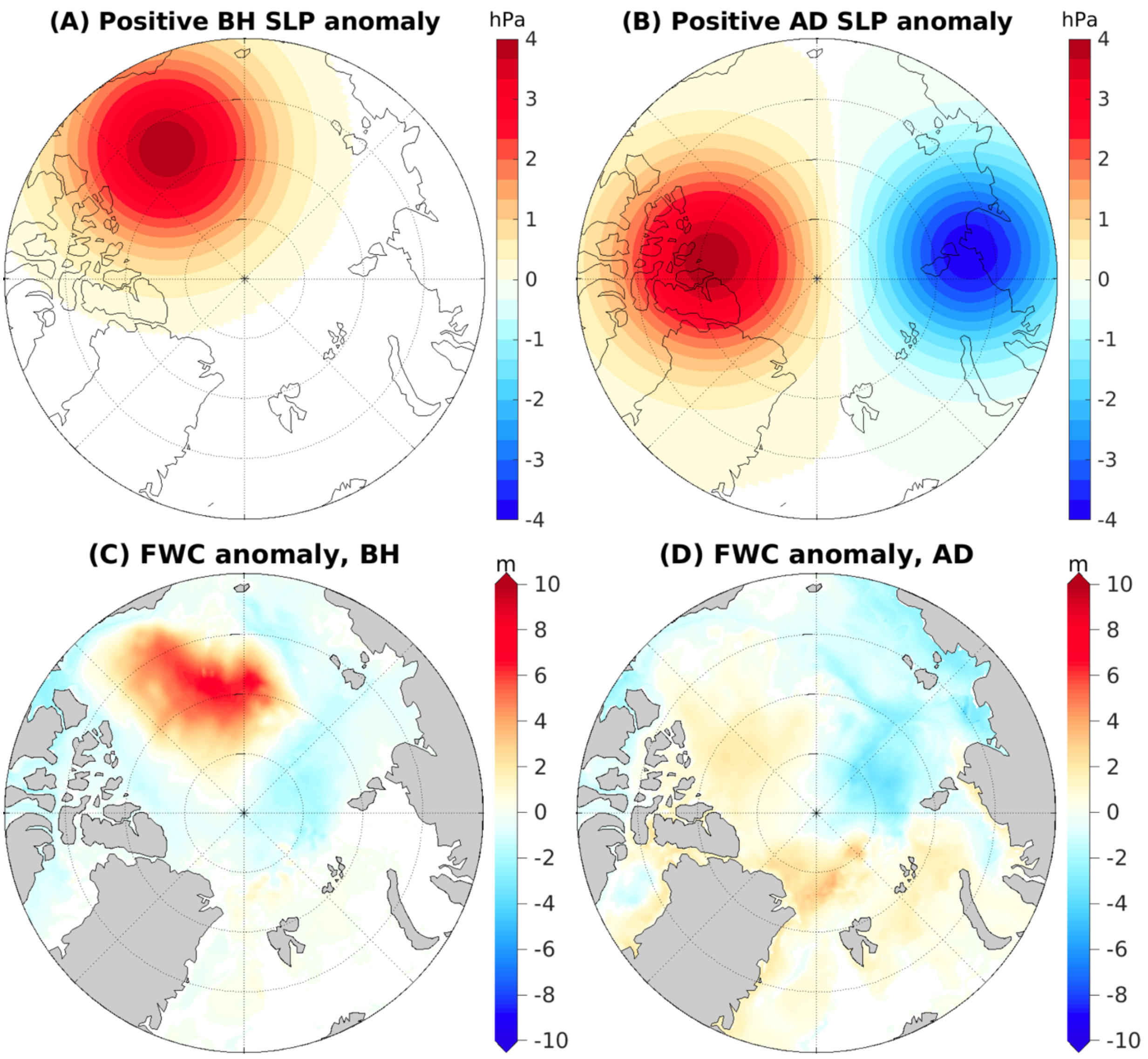

Fig. S11. Impact of the spatial positioning of sea level pressure (SLP) anomalies on freshwater content (FWC). (**A** and **B**) SLP anomalies representing the positive phase of (A) the Beaufort High (BH) and (B) the Arctic Dipole (AD). Wind associated with these SLP anomalies are used in the respective idealized wind-perturbation experiments (see Method). (**C** and **D**) The obtained response of FWC to the corresponding (C) BH and (D) AD wind forcings.

REFERENCES AND NOTES

1. M. M. Holland, C. M. Bitz, Polar amplification of climate change in coupled models. *Clim. Dyn.* **21**, 221–232 (2003).

2. M. Rantanen, A. Y. Karpechko, A. Lipponen, K. Nordling, O. Hyvärinen, K. Ruosteenoja, T. Vihma, A. Laaksonen, The Arctic has warmed nearly four times faster than the globe since 1979. *Commun. Earth Environ.* **3**, 168 (2022).

3. R. Kwok, Arctic sea ice thickness, volume, and multiyear ice coverage: Losses and coupled variability (1958–2018). *Environ. Res. Lett.* **13**, 105005 (2018).

4. Intergovernmental Panel on Climate Change, in *Climate Change 2021: The Physical Science Basis* (Cambridge Univ. Press, 2023), pp. 1211–1362.

5. K. A. Giles, S. W. Laxon, A. L. Ridout, D. J. Wingham, S. Bacon, Western Arctic Ocean freshwater storage increased by wind-driven spin-up of the Beaufort Gyre. *Nat. Geosci.* **5**, 194–197 (2012).

6. J. Morison, R. Kwok, C. Peralta-Ferriz, M. Alkire, I. Rigor, R. Andersen, M. Steele, Changing Arctic Ocean freshwater pathways. *Nature* **481**, 66–70 (2012).

7. A. Proshutinsky, R. Krishfield, J. M. Toole, M.-L. Timmermans, W. Williams, S. Zimmermann, M. Yamamoto-Kawai, T. W. K. Armitage, D. Dukhovskoy, E. Golubeva, G. E. Manucharyan, G. Platov, E. Watanabe, T. Kikuchi, S. Nishino, M. Itoh, S.-H. Kang, K.-H. Cho, K. Tateyama, J. Zhao, Analysis of the Beaufort Gyre freshwater content in 2003–2018. *J. Geophys. Res. Oceans* **124**, 9658–9689 (2019).

8. M.-L. Timmermans, J. M. Toole, The Arctic Ocean's Beaufort Gyre. *Ann. Rev. Mar. Sci.* **15**, 223–248 (2023).

9. I. V. Polyakov, A. V. Pnyushkov, M. B. Alkire, I. M. Ashik, T. M. Baumann, E. C. Carmack, I. Goszczko, J. Guthrie, V. V. Ivanov, T. Kanzow, R. Krishfield, R. Kwok, A. Sundfjord, J.

Morison, R. Rember, A. Yulin, Greater role for Atlantic inflows on sea-ice loss in the Eurasian Basin of the Arctic Ocean. *Science* **356**, 285–291 (2017).

10. O. Arzel, T. Fichefet, H. Goosse, J.-L. Dufresne, Causes and impacts of changes in the Arctic freshwater budget during the twentieth and twenty-first centuries in an AOGCM. *Clim. Dyn.* **30**, 37–58 (2008).

11. J. Zhang, W. Weijer, M. Steele, W. Cheng, T. Verma, M. Veneziani, Labrador Sea freshening linked to Beaufort Gyre freshwater release. *Nat. Commun.* **12**, 1229 (2021).

12. W. Weijer, T. Haine, A. Siddiqui, W. Cheng, M. Veneziani, P. Kurtakoti, Interactions between the Arctic Mediterranean and the Atlantic Meridional Overturning Circulation: A review. *Oceanography* **35**, 118–127 (2022).

13. V. Ivanov, V. Alexeev, N. V. Koldunov, I. Repina, A. B. Sandø, L. H. Smedsrud, A. Smirnov, Arctic Ocean heat impact on regional ice decay: A suggested positive feedback. *J. Phys. Oceanogr.* **46**, 1437–1456 (2016).

14. I. V. Polyakov, T. P. Rippeth, I. Fer, M. B. Alkire, T. M. Baumann, E. C. Carmack, R. Ingvaldsen, V. V. Ivanov, M. Janout, S. Lind, L. Padman, A. V. Pnyushkov, R. Rember, Weakening of cold halocline layer exposes sea ice to oceanic heat in the eastern Arctic Ocean. *J. Clim.* **33**, 8107–8123 (2020).

15. R. B. Ingvaldsen, K. M. Assmann, R. Primicerio, M. Fossheim, I. V. Polyakov, A. V. Dolgov, Physical manifestations and ecological implications of Arctic Atlantification. *Nat. Rev. Earth Environ.* **2**, 874–889 (2021).

16. W.-J. von Appen, A. M. Waite, M. Bergmann, C. Bienhold, O. Boebel, A. Bracher, B. Cisewski, J. Hagemann, M. Hoppema, M. H. Iversen, C. Konrad, T. Krumpen, N. Lochthofen, K. Metfies, B. Niehoff, E.-M. Nöthig, A. Purser, I. Salter, M. Schaber, D. Scholz, T. Soltwedel, S. Torres-Valdes, C. Wekerle, F. Wenzhöfer, M. Wietz, A. Boetius, Sea-ice derived meltwater stratification slows the biological carbon pump: Results from continuous observations. *Nat. Commun.* **12**, 7309 (2021).

17. S. Chatterjee, R. P. Raj, L. Bertino, Ø. Skagseth, M. Ravichandran, O. M. Johannessen, Role of Greenland Sea gyre circulation on Atlantic Water temperature variability in the Fram Strait. *Geophys. Res. Lett.* **45**, 8399–8406 (2018).

18. M. L. Timmermans, J. Marshall, Understanding Arctic Ocean circulation: A review of ocean dynamics in a changing climate. *J. Geophys. Res. Oceans* **125**, e2018JC014378 (2020).

19. M. Muilwijk, M. Ilicak, S. B. Cornish, S. Danilov, R. Gelderloos, R. Gerdes, V. Haid, T. W. N. Haine, H. L. Johnson, Y. Kostov, T. Kovács, C. Lique, J. M. Marson, P. G. Myers, J. Scott, L. H. Smedsrud, C. Talandier, Q. Wang, Arctic Ocean response to Greenland Sea wind anomalies in a suite of model simulations. *J. Geophys. Res. Oceans* **124**, 6286–6322 (2019).

20. Q. Wang, C. Wekerle, X. Wang, S. Danilov, N. Koldunov, D. Sein, D. Sidorenko, W. J. von Appen, T. Jung, Intensification of the Atlantic Water supply to the Arctic Ocean through Fram Strait induced by Arctic sea ice decline. *Geophys. Res. Lett.* **47**, e2019GL086682 (2020).

21. Q. Wang, Q. Shu, S. Wang, A. Beszczynska-Moeller, S. Danilov, L. Steur, T. W. N. Haine, M. Karcher, C. M. Lee, P. G. Myers, I. V. Polyakov, C. Provost, Ø. Skagseth, G. Spreen, R. Woodgate, A review of Arctic–Subarctic Ocean linkages: Past changes, mechanisms, and future projections. *Ocean-Land-Atmos. Res.* **2**, 0013 (2023).

22. B. Wu, J. Wang, J. E. Walsh, Dipole anomaly in the winter Arctic atmosphere and its association with sea ice motion. *J. Clim.* **19**, 210–225 (2006).

23. I. V. Polyakov, R. B. Ingvaldsen, A. V. Pnyushkov, U. S. Bhatt, J. A. Francis, M. Janout, R. Kwok, Ø. Skagseth, Fluctuating Atlantic inflows modulate Arctic atlantification. *Science* **381**, 972–979 (2023).

24. S. Lind, R. B. Ingvaldsen, T. Furevik, Arctic warming hotspot in the northern Barents Sea linked to declining sea-ice import. *Nat. Clim. Chang.* **8**, 634–639 (2018).

25. H. Asbjørnsen, M. Årthun, Ø. Skagseth, T. Eldevik, Mechanisms underlying recent Arctic atlantification. *Geophys. Res. Lett.* **47**, e2020GL088036 (2020).

26. Ø. Skagseth, T. Eldevik, M. Årthun, H. Asbjørnsen, V. S. Lien, L. H. Smedsrud, Reduced efficiency of the Barents Sea cooling machine. *Nat. Clim. Chang.* **10**, 661–666 (2020).

27. Q. Shu, Q. Wang, Z. Song, F. Qiao, The poleward enhanced Arctic Ocean cooling machine in a warming climate. *Nat. Commun.* **12**, 2966 (2021).

28. D. W. J. Thompson, J. M. Wallace, The Arctic oscillation signature in the wintertime geopotential height and temperature fields. *Geophys. Res. Lett.* **25**, 1297–1300 (1998).

29. P. Lin, R. S. Pickart, H. Heorton, M. Tsamados, M. Itoh, T. Kikuchi, Recent state transition of the Arctic Ocean's Beaufort Gyre. *Nat. Geosci.* **16**, 485–491 (2023).

30. Q. Wang, S. Danilov, A synthesis of the upper Arctic Ocean circulation during 2000–2019: Understanding the roles of wind forcing and sea ice decline. *Front. Mar. Sci.* **9**, 863204 (2022).

31. R. A. Woodgate, C. Peralta-Ferriz, Warming and freshening of the Pacific inflow to the Arctic from 1990-2019 implying dramatic shoaling in Pacific Winter Water ventilation of the Arctic water column. *Geophys. Res. Lett.* **48**, e2021GL092528 (2021).

32. M. Steele, J. Morison, W. Ermold, I. Rigor, M. Ortmeyer, K. Shimada, Circulation of summer Pacific halocline water in the Arctic Ocean. *J. Geophys. Res. Oceans* **109**, C02027 (2004).

33. J. Morison, M. Steele, T. Kikuchi, K. Falkner, W. Smethie, Relaxation of central Arctic Ocean hydrography to pre-1990s climatology. *Geophys. Res. Lett.* **33**, L17604 (2006).

34. A. Proshutinsky, D. Dukhovskoy, M. Timmermans, R. Krishfield, J. L. Bamber, Arctic circulation regimes. *Philos. Trans. R. Soc. London Ser. A Math. Phys. Eng. Sci.* **373**, 20140160 (2015).

35. Q. Wang, C. Wekerle, S. Danilov, D. Sidorenko, N. Koldunov, D. Sein, B. Rabe, T. Jung, Recent sea ice decline did not significantly increase the total liquid freshwater content of the Arctic Ocean. *J. Clim.* **32**, 15–32 (2019).

36. S. Wang, Q. Wang, Q. Shu, Z. Song, G. Lohmann, S. Danilov, F. Qiao, Nonmonotonic change of the Arctic Ocean freshwater storage capability in a warming climate. *Geophys. Res. Lett.* **48**, e2020GL090951 (2021).

37. W. Maslowski, B. Newton, P. Schlosser, A. Semtner, D. Martinson, Modeling recent climate variability in the Arctic Ocean. *Geophys. Res. Lett.* **27**, 3743–3746 (2000).

38. X. Zhang, M. Ikeda, J. E. Walsh, Arctic sea ice and freshwater changes driven by the atmospheric leading mode in a coupled sea ice–ocean model. *J. Clim.* **16**, 2159–2177 (2003).

39. L. Oziel, J. Sirven, J.-C. Gascard, The Barents Sea frontal zones and water masses variability (1980–2011). *Ocean Sci.* **12**, 169–184 (2016).

40. B. I. Barton, Y.-D. Lenn, C. Lique, Observed atlantification of the Barents Sea causes the Polar Front to limit the expansion of winter sea ice. *J. Phys. Oceanogr.* **48**, 1849–1866 (2018).

41. V. S. Lien, P. Schlichtholz, Ø. Skagseth, F. B. Vikebø, Wind-driven atlantic water flow as a direct mode for reduced Barents Sea ice cover. *J. Clim.* **30**, 803–812 (2017).

42. F. O. Heukamp, L. Aue, Q. Wang, M. Ionita, T. Kanzow, C. Wekerle, A. Rinke, Cyclones modulate the control of the North Atlantic Oscillation on transports into the Barents Sea. *Commun. Earth Environ.* **4**, 324 (2023).

43. Y. Liu, Y. He, Cold season Arctic strong cyclones enhance Atlantification of the Arctic Ocean. *Environ. Res. Lett.* **18**, 114049 (2023).

44. I. V. Polyakov, T. P. Rippeth, I. Fer, T. M. Baumann, E. C. Carmack, V. V. Ivanov, M. Janout, L. Padman, A. V. Pnyushkov, R. Rember, Intensification of near-surface currents and shear in the Eastern Arctic Ocean. *Geophys. Res. Lett.* **47**, e2020GL089469 (2020).

45. P. Duarte, A. Sundfjord, A. Meyer, S. R. Hudson, G. Spreen, L. H. Smedsrud, Warm Atlantic water explains observed sea ice melt rates north of Svalbard. *J. Geophys. Res. Oceans* **125**, e2019JC015662 (2020).

46. M. Årthun, T. Eldevik, L. H. Smedsrud, The role of Atlantic heat transport in future Arctic winter sea ice loss. *J. Clim.* **32**, 3327–3341 (2019).

47. X. Zhang, H. Tang, J. Zhang, J. E. Walsh, E. L. Roesler, B. Hillman, T. J. Ballinger, W. Weijer, Arctic cyclones have become more intense and longer-lived over the past seven decades. *Commun. Earth Environ.* **4**, 348 (2023).

48. G. W. K. Moore, A. Schweiger, J. Zhang, M. Steele, Collapse of the 2017 winter Beaufort High: A response to thinning sea ice? *Geophys. Res. Lett.* **45**, 2860–2869 (2018).

49. J. S. Kenigson, M.-L. Timmermans, Arctic cyclone activity and the Beaufort High. *J. Clim.* **34**, 4119–4127 (2021).

50. Z. Long, W. Perrie, M. Zhang, Y. Liu, Responses of Atlantic water inflow through Fram Strait to Arctic storms. *Geophys. Res. Lett.* **51**, e2023GL107777 (2024).

51. J. A. Screen, I. Simmonds, The central role of diminishing sea ice in recent Arctic temperature amplification. *Nature* **464**, 1334–1337 (2010).

52. M. C. Serreze, R. G. Barry, Processes and impacts of Arctic amplification: A research synthesis. *Glob. Planet. Change* **77**, 85–96 (2011).

53. Q. Shu, Q. Wang, M. Årthun, S. Wang, Z. Song, M. Zhang, F. Qiao, Arctic Ocean Amplification in a warming climate in CMIP6 models. *Sci. Adv.* **8**, eabn9755 (2022).

54. J. Morison, R. Kwok, S. Dickinson, R. Andersen, C. Peralta-Ferriz, D. Morison, I. Rigor, S. Dewey, A. J. Guthrie, The cyclonic mode of Arctic Ocean circulation. *J. Phys. Oceanogr.* **51**, 1053–1075 (2021).

55. E. C. Carmack, R. W. Macdonald, R. G. Perkin, F. A. McLaughlin, R. J. Pearson, Evidence for warming of Atlantic water in the Southern Canadian Basin of the Arctic Ocean: Results from the Larsen-93 Expedition. *Geophys. Res. Lett.* **22**, 1061–1064 (1995).

56. J. Morison, M. Steele, R. Andersen, Hydrography of the upper Arctic Ocean measured from the nuclear submarine U.S.S. *Pargo*. *Deep Sea Res. 1 Oceanogr. Res. Pap.* **45**, 15–38 (1998).

57. M. Steele, T. Boyd, Retreat of the cold halocline layer in the Arctic Ocean. *J. Geophys. Res. Oceans* **103**, 10419–10435 (1998).

58. R. R. Dickson, T. J. Osborn, J. W. Hurrell, J. Meincke, J. Blindheim, B. Adlandsvik, T. Vinje, G. Alekseev, W. Maslowski, The Arctic Ocean response to the North Atlantic oscillation. *J. Clim.* **13**, 2671–2696 (2000).

59. H. L. Johnson, S. B. Cornish, Y. Kostov, E. Beer, C. Lique, Arctic Ocean freshwater content and its decadal memory of sea-level pressure. *Geophys. Res. Lett.* **45**, 4991–5001 (2018).

60. H. Tsujino, S. Urakawa, H. Nakano, R. J. Small, W. M. Kim, S. G. Yeager, G. Danabasoglu, T. Suzuki, J. L. Bamber, M. Bentsen, C. W. Böning, A. Bozec, E. P. Chassignet, E. Curchitser, F. Boeira Dias, P. J. Durack, S. M. Griffies, Y. Harada, M. Ilicak, S. A. Josey, C. Kobayashi, S. Kobayashi, Y. Komuro, W. G. Large, J. Le Sommer, S. J. Marsland, S. Masina, M. Scheinert, H. Tomita, M. Valdivieso, D. Yamazaki, JRA-55 based surface dataset for driving ocean–sea-ice models (JRA55-do). *Ocean Model.* **130**, 79–139 (2018).

61. Q. Wang, C. Wekerle, S. Danilov, X. Wang, T. Jung, A 4.5g km resolution Arctic Ocean simulation with the global multi-resolution model FESOM 1.4. *Geosci. Model Dev.* **11**, 1229–1255 (2018).

62. S. Danilov, Q. Wang, R. Timmermann, N. Iakovlev, D. Sidorenko, M. Kimmritz, T. Jung, J. Schröter, Finite-Element Sea Ice Model (FESIM), version 2. *Geosci. Model Dev.* **8**, 1747–1761 (2015).

63. M. Steele, R. Morley, W. Ermold, PHC: A global ocean hydrography with a high-quality Arctic Ocean. *J. Clim.* **14**, 2079–2087 (2001).

64. Q. Wang, Q. Shu, A. Bozec, E. P. Chassignet, P. G. Fogli, B. Fox-Kemper, A. M. Hogg, D. Iovino, A. E. Kiss, N. Koldunov, J. Le Sommer, Y. Li, P. Lin, H. Liu, I. Polyakov, P. Scholz, D. Sidorenko, S. Wang, X. Xu, Impact of increased resolution on Arctic Ocean simulations in

Ocean Model Intercomparison Project phase 2 (OMIP-2). *Geosci. Model Dev.* **17**, 347–379 (2024).

65. A. Y. Proshutinsky, M. A. Johnson, Two circulation regimes of the wind-driven Arctic Ocean. *J. Geophys. Res. Oceans* **102**, 12493–12514 (1997).

66. A. Beszczynska-Möller, E. Fahrbach, U. Schauer, E. Hansen, Variability in Atlantic water temperature and transport at the entrance to the Arctic Ocean, 1997–2010. *ICES J. Mar. Sci.* **69**, 852–863 (2012).

67. K. von Schuckmann, P.-Y. Le Traon, N. Smith, A. Pascual, P. Brasseur, K. Fennel, S. Djavidnia, S. Aaboe, E. A. Fanjul, E. Autret, L. Axell, R. Aznar, M. Benincasa, A. Bentamy, F. Boberg, R. Bourdallé-Badie, B. B. Nardelli, V. E. Brando, C. Bricaud, L.-A. Breivik, R. J. W. Brewin, A. Capet, A. Ceschin, S. Ciliberti, G. Cossarini, M. de Alfonso, A. de Pascual Collar, J. de Kloe, J. Deshayes, C. Desportes, M. Drévillon, Y. Drillet, R. Droghei, C. Dubois, O. Embury, H. Etienne, C. Fratianni, J. G. Lafuente, M. G. Sotillo, G. Garric, F. Gasparin, R. Gerin, S. Good, J. Gourrion, M. Grégoire, E. Greiner, S. Guinehut, E. Gutknecht, F. Hernandez, O. Hernandez, J. Høyer, L. Jackson, S. Jandt, S. Josey, M. Juza, J. Kennedy, Z. Kokkini, G. Korres, M. Kõuts, P. Lagemaa, T. Lavergne, B. le Cann, J.-F. Legeais, B. Lemieux-Dudon, B. Levier, V. Lien, I. Maljutenko, F. Manzano, M. Marcos, V. Marinova, S. Masina, E. Mauri, M. Mayer, A. Melet, F. Mélin, B. Meyssignac, M. Monier, M. Müller, S. Mulet, C. Naranjo, G. Notarstefano, A. Paulmier, B. P. Gomez, I. P. Gonzalez, E. Peneva, C. Perruche, K. A. Peterson, N. Pinardi, A. Pisano, S. Pardo, P.-M. Poulain, R. P. Raj, U. Raudsepp, M. Ravdas, R. Reid, M.-H. Rio, S. Salon, A. Samuelsen, M. Sammartino, S. Sammartino, A. B. Sandø, R. Santoleri, S. Sathyendranath, J. She, S. Simoncelli, C. Solidoro, A. Stoffelen, A. Storto, T. Szerkely, S. Tamm, S. Tietsche, J. Tinker, J. Tintore, A. Trindade, D. van Zanten, L. Vandenbulcke, A. Verhoef, N. Verbrugge, L. Viktorsson, K. von Schuckmann, S. L. Wakelin, A. Zacharioudaki, H. Zuo, Copernicus Marine Service Ocean State Report. *J. Oper. Oceanogr.* **11**, S1–S142 (2018).

68. K. Aagaard, E. C. Carmack, The role of sea ice and other fresh water in the Arctic circulation. *J. Geophys. Res. Oceans* **94**, 14485–14498 (1989).

69. A. Schweiger, R. Lindsay, J. Zhang, M. Steele, H. Stern, R. Kwok, Uncertainty in modeled Arctic sea ice volume. *J. Geophys. Res. Oceans* **116**, C00D06 (2011).

70. F. Fetterer, K. Knowles, W. N. Meier, M. Savoie, A. K. Windnagel, Sea Ice Index, version 3, National Snow Ice Data Center (2017).

71. W.-J. Von Appen, U. Schauer, T. Hattermann, A. Beszczynska-Möller, Seasonal cycle of mesoscale instability of the West Spitsbergen Current. *J. Phys. Oceanogr.* **46**, 1231–1254 (2016).
